# Abrupt declines in fish productivity often precede stock collapses in marine fisheries

**DOI:** 10.1126/sciadv.aed7911

**Published:** 2026-09-02

**Authors:** Mathieu Pélissié, Vincent Devictor, Olaf P. Jensen, Vasilis Dakos

**Affiliations:** ^1^ Institut des Sciences de l’Évolution de Montpellier (ISEM), Univ. Montpellier, CNRS, IRD, Montpellier, France.; ^2^ Department of Biology, University of Padova, Padova, Italy.; ^3^ Center for Limnology, University of Wisconsin–Madison, Madison, WI, USA.; ^4^ Cornell Biological Field Station and Ashley School of Global Development and the Environment, Cornell University, Ithaca, NY, USA.

## Abstract

Marine life is under multiple pressures, including climate change and overfishing, which can threaten fishery sustainability especially when it results in large, abrupt, and persistent shifts in fish productivity. Reports of abrupt shifts in marine systems are not uncommon, but a global assessment of their occurrence and drivers is seriously lacking. Here, we systematically classified the temporal dynamics of fish stock productivity from agency-assessed fisheries worldwide. We detected productivity abrupt shift in more than a quarter of the 315 fish stocks analyzed. Abrupt declines were markedly overrepresented in marine regions with highest warming rates, while abrupt increases were more likely under lower fishing intensity. We also demonstrate that abrupt productivity declines preceded stock collapses by about a decade in a quarter of the stocks that shifted. Overall, our results highlight the importance of considering productivity abrupt shifts to prevent an increasing risk of fish population collapse in warming oceans.

## INTRODUCTION

Marine ecosystems are facing intense anthropogenic pressure (*1*, *2*), putting the sustainability of marine species along with the food security and economies of many coastal human populations at risk (*3*). Although the capture of wild fish from the oceans has been maintained at high levels since its peak in the 1990s (*4*), it does not imply that all stocks are sustainably exploited (*3*). Despite the overall success of some management strategies (*5*, *6*), overexploitation and stock collapses are still major threats (*2*) with no guarantee that recent improvements in fishery sustainability (*7*) can be maintained in the face of climate change.

One particular challenge for fisheries management is the ability to avoid abrupt, high-amplitude, and persistent changes of exploited stocks, which undermine sustainability goals (*8*, *9*). Although the existence of such so-called regime shifts has long been documented in fisheries (*10*–*12*), these events have yet to be broadly integrated within stock assessment and management (*13*, *14*). Formal definitions of regime shifts often involve synchronous change across multiple trophic levels from fish to phytoplankton, or permanent abrupt changes in pelagic or benthic community composition along with changes in climatic and oceanic components (*15*–*17*). Although an abrupt shift in any ecological variable is not necessarily a regime shift, it has been argued that abrupt shifts recorded at the level of fish populations could be indicative of putative regime shifts affecting whole ecosystems (*18*–*21*). In fisheries, examples of once plentiful stocks that crashed to very low levels are not uncommon, notably with the case of the Peruvian anchoveta (*22*), North Atlantic cod (*23*), or Western Atlantic bluefin tuna (*24*). However, beyond these emblematic examples that might have caught most attention, the prevalence of regime shifts in global fisheries in recent years remains mostly underestimated.

Accounting for regime shifts within fisheries management can be hindered by two major limitations. First, they are not straightforward to detect. The search for regime shifts in time series often relies on the sole use of breakpoint detection algorithms without relevant alternative non-abrupt models (*25*), which can limit the confidence in the conclusions (*26*). Second, mechanisms and feedbacks that could give rise to regime shift dynamics are usually overlooked in stock assessment models, despite evidence that regime shift responses are frequently a better descriptor of stock dynamics (*27*). Attempts to account for regime shifts and nonstationarity remain scarce and insufficient to improve current practices (*9*, *13*). Instead, the usual standard for evaluating the sustainability of management strategies relies on fixed thresholds—related to biological reference points like minimal biomass or maximal fishing mortality—to trigger actions (*28*), despite recent efforts to introduce more dynamic reference points (*29*, *30*).

Although the notions of regime shifts and stock collapses appear strongly connected, they are not equivalent. Regime shifts are usually characterized by an abrupt temporal trajectory with a final state persistent in time (*31*). In contrast, stock collapses relate to stock depletion based on fixed thresholds related to stock size or catch (*32*) that give no indication about the circumstances preceding the collapse nor the persistence of the collapse state. For instance, it has been shown that abrupt shifts from high to low surplus production levels—a proxy for stock productivity corresponding to the change in abundance in the absence of fishing (*33*)—can happen quite regularly and were unrelated to abundance levels for a substantial proportion of (but not all) stocks (*27*). Although this finding implies that abrupt shifts in productivity do not necessarily lead to stock collapse, the relationship between the two phenomena has not been systematically explored. In addition to not fully understanding the consequences of abrupt shifts in productivity, we also poorly understand what causes them. The relative contributions of climate and exploitation to stock collapse have been investigated only for a limited number of data-rich stocks (*34*, *35*). Broader syntheses of stock collapse mostly focus on a single risk factor at a time, either fishing intensity (*36*), climate change (*37*), or life history (*38*) but rarely all pressures together (*39*). Some regional or basin-scale assessments of abrupt shifts and potential regime shifts have also been conducted, but most of them relied on recruitment dynamics (*40*–*42*). Overall, we lack a global overview of the prevalence of abrupt shifts in fisheries productivity, along with an understanding of their drivers, and potential link with stock collapses.

To address this gap, in this study, we estimate abrupt shifts in fish productivity—defined as large and rapid changes relative to background fluctuations—that could serve as hallmarks to potential regime shifts in marine fish stocks globally. Here, we systematically classify the dynamics of 315 productivity time series, estimated from assessments of marine fish stocks from around the world, to address the following questions: (i) How prevalent are productivity abrupt shifts (PASs) and how are they distributed in space and time? (ii) Are PASs related to species life history, environmental conditions, or fishing pressure? (iii) Are PASs associated with stock collapses?

## RESULTS

### PAS prevalence and distribution

**Fig. 1.**
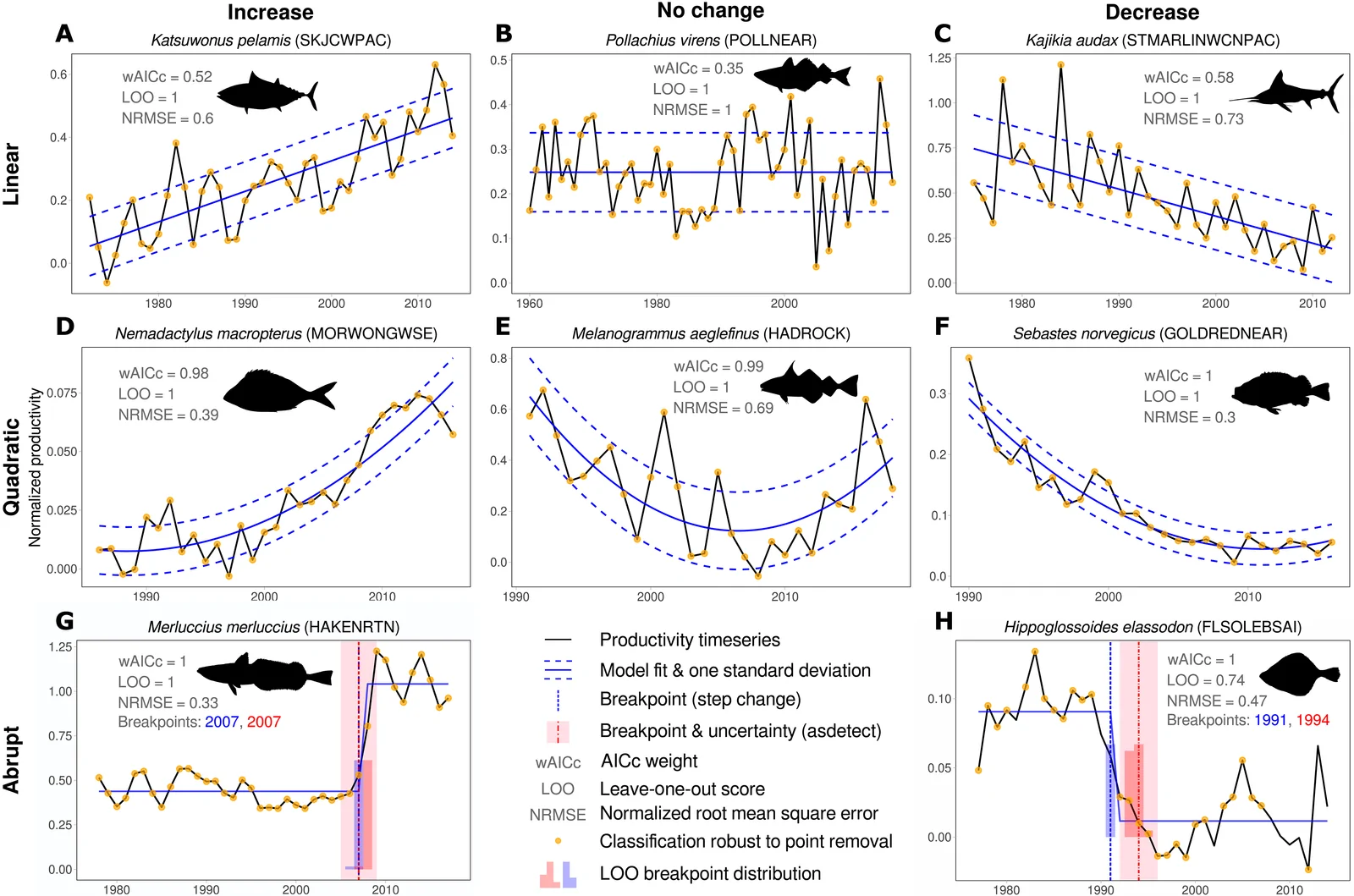
Examples of productivity time series classified into trajectory types. Classification is based on shape and trend. Some time series were best described by either linear (**A** to **C**), quadratic (**D** to **F**), or abrupt trajectories (**G** and **H**) and overall trend either positive [(A), (D), and (G)] or negative [(C), (F), and (H)]. No change is a type of shape that corresponds to a trajectory without trend [(B) and (E)]. Each panel shows fish stock productivity time series normalized by the average stock biomass (black line) with the best model fit (solid blue line) and SD (dashed lines) following the classification procedure detailed in Materials and Methods. Three classification quality scores are specified for each time series: AICc weight (wAICc), leave-one-out score (LOO), and normalized root mean square error (NRMSE). Time points that if removed in the LOO process would result in the same shape are highlighted by orange dots. For abrupt trajectories [(G) and (H)], the location of breakpoints is indicated by vertical lines, the pink background corresponds to the breakpoint uncertainty for the asdetect method, and the distribution of breakpoint locations from LOO time series are represented by color bars. Note that scales are not the same for the different panels. Species name and stock ID are also displayed. Fish silhouettes were obtained from PhyloPic images (www.phylopic.org/) under CC0 1.0 or PDM 1.0 licenses.

We classified 315 fish stock productivity time series into basic trajectory types based on shape and trend and found all types of trajectories over the period 1950–2020 (Fig. 1). Quality scores indicated that selected models, especially for abrupt and quadratic trajectories, performed well and were robust to the removal of individual data points (Fig. 1 and fig. S1). Still, the variance explained by the models remained low given the intrinsically high variability of productivity time series (fig. S1). Some well-documented previously characterized abrupt shifts were correctly captured by the classification, notably for the North Atlantic cod in the 1990s, Japanese sardine in the late 1980s, and Pacific herring in the 1960s (fig. S2). The full list of trajectories is available in the Supplementary Materials (table S1).

**Fig. 2.**
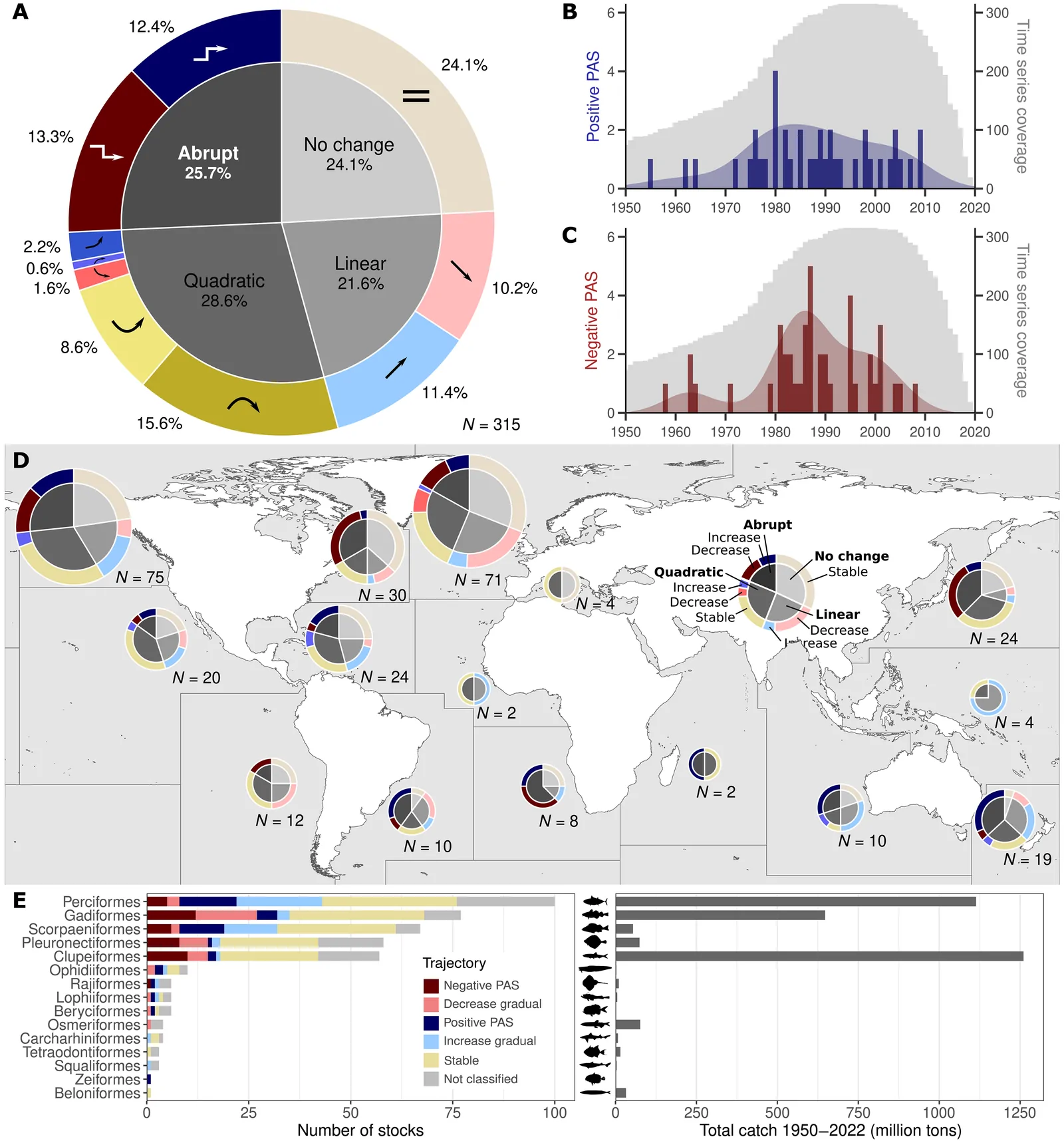
Distribution of productivity trajectory types in space, time, and taxonomic groups. Overview and spatial distribution of all productivity trajectory types alongside the temporal distribution of PASs. (**A**) Overall proportion of all productivity trajectory types, with the inner pie chart indicating trajectory shape and the outer layer specifying the trend. (**B** and **C**) Temporal distribution of positive (B) and negative PASs (C) (color bars and density) with, in gray, the coverage (i.e., number of stocks with data available in each year) of the time series classified. (**D**) Spatial distribution of all trajectory types by FAO major fishing areas. The size of the charts is indicative of the number of stocks, which are also displayed. (**E**) Productivity trajectories by taxonomic order and total catch worldwide between 1950 and 2022 based on FAO data. Stocks that were not classified (e.g., length below 25 years) are in gray. Fish silhouettes were obtained from PhyloPic images (www.phylopic.org/) under CC0 1.0 or PDM 1.0 licenses.

Globally, PASs were found for more than a quarter of stocks (25.7%, *N* = 81), with roughly equal numbers of negative (13.3%, *N* = 42) and positive (12.4%, *N* = 39) PASs (Fig. 2A). The other types of trajectories (quadratic, linear, and no change) were found in similar proportions. Balanced proportions between different trajectory types were also found at the scale of FAO major fishing areas (Fig. 2D), with no significant difference across areas (chi-square test, *P* = 0.32). The direction of shifts was however unevenly distributed in space (chi-square test, *P* = 0.02), with regions like the North-West Atlantic and North-West Pacific comprising a higher proportion of negative PASs (30 and 29%, respectively), whereas the region with most positive PASs (32%) was the South-West Pacific. Similar patterns were found using large marine ecosystems (LMEs) as grouping areas (fig. S3). Considering taxonomic groups, we found significant differences in the proportions of PASs against non-abrupt trajectories across the five most numerous orders in number of stocks (chi-square test, *P* = 0.01), with notably a higher proportion of positive PASs in Perciformes and of negative PASs in Clupeiformes (Fig. 2E).

Over time, the occurrence of positive PASs was spread from the 1960s to the 2010s, and the distribution did not differ significantly from the coverage of time series (Fig. 2B; Kolmogorov-Smirnov test, *P* = 0.32). However, the distribution of negative PASs differed from coverage (Kolmogorov-Smirnov test, *P* = 0.04) and tended to cluster during the 1980s (Fig. 2C), before the maximum of time series available was reached between 1994 and 2000. The observation of PASs being less frequent after 2010 could arise from a lack of available data along with the difficulty of detecting very recent shifts.

### Drivers of PAS

**Fig. 3.**
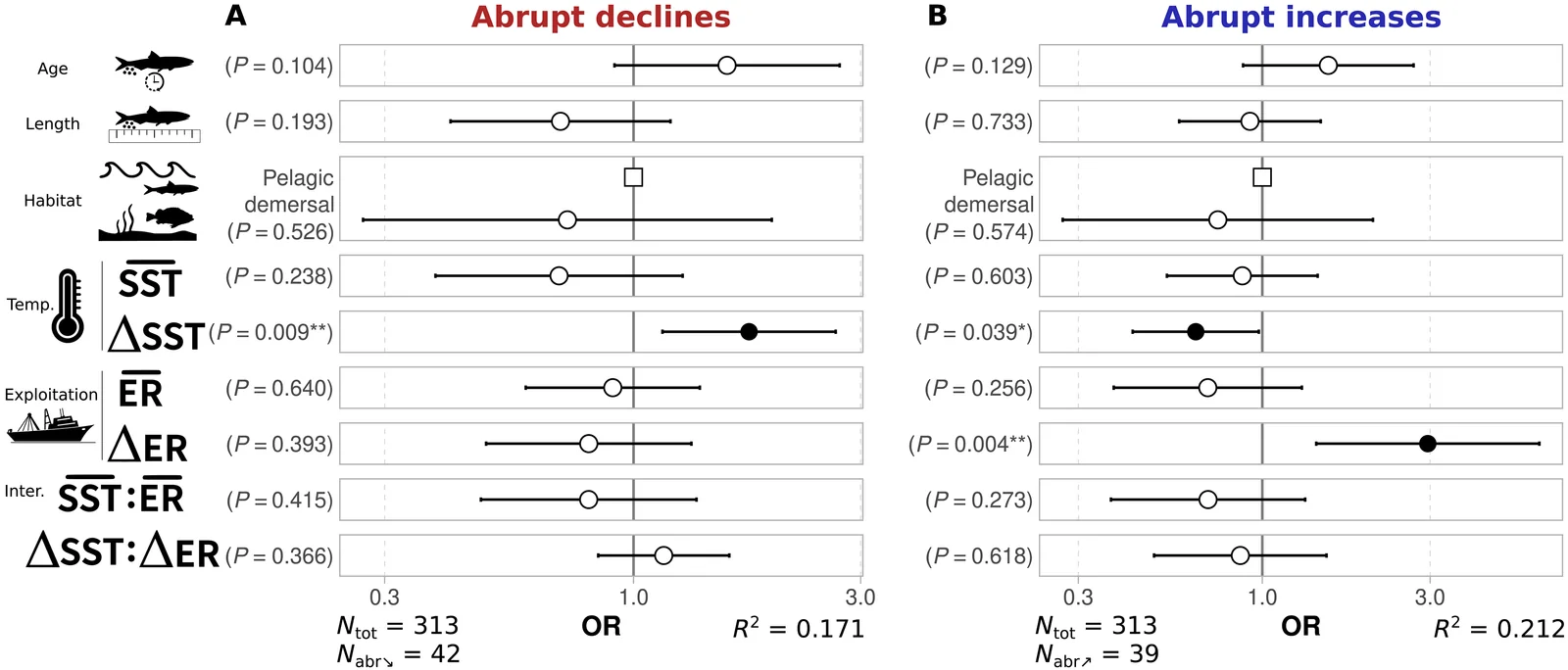
Quantification of the drivers of PAS. GAMM odds ratios for models accounting for negative (**A**) and positive (**B**) PASs against all other trajectories. Positive estimates mean that PASs are more often found for larger values of the variable and conversely. Dependent variables are arranged from top to bottom with age at maturity, length at maturity, main habitat (pelagic as reference category), mean ($\bar{\mathrm{SST}}$) and linear trend (ΔSST) in *SST*, and mean ($\bar{\mathrm{ER}}$) and linear trend (ΔER) in *ER*. The interactions between mean *SST* and *ER* and linear trends in *SST* and *ER* were also tested. Mean and trend in *SST* and *ER* were computed from the first year of productivity available up to the shift if any was detected. Odd ratios (OR, circles) with confidence intervals at 95% (horizontal bars) are presented on a logarithmic scale, and significant odd ratios are indicated with filled circles. Model fits were estimated with McFadden’s *R*^2^, which correspond to the pseudo-R^2^ for logistic regression models. All numeric variables were standardized.

We tested which drivers among life history, environment (sea surface temperature, *SST*), and fishing intensity (exploitation rate, *ER*) were more related to the occurrence of PAS using generalized additive mixed models (GAMMs) to account for phylogeny and spatial location. We found that the main drivers of PAS differed between negative and positive PASs. Negative PASs were mostly influenced by environmental conditions like higher trends in *SST* occurring before the shift (*odds ratio* = 1.75, *CI*_*95%*_ = [1.15, 2.66], *P* = 0.009; Fig. 3A) and marginally by lower average *SST* (*odds ratio* = 1.57, *CI*_*95%*_ = [0.91, 2.71], *P* = 0.104; Fig. 3A) compared to other trajectories. Positive PASs were influenced by fishing intensity and life history and were associated with higher trends in *ER* (*odds ratio* = 2.95, *CI*_*95%*_ = [1.42, 6.13], *P* = 0.004; Fig. 3B), lower trends in *SST* (*odds ratio* = 0.65, *CI*_*95%*_ = [0.43, 0.98], *P* = 0.039; Fig. 3B), and marginally with higher age at maturity (*odds ratio* = 1.54, *CI*_*95%*_ = [0.88, 2.69], *P* = 0.129; Fig. 3B). No interaction between *SST* and *ER* was found, either in terms of average values or trends.

As both models for negative and positive PASs explained between 17 and 21% of the total variance (McFadden’s *R*^2^ for logistic regression; Fig. 3), the occurrence of PASs was only partly explained by the variables selected. But the identity and relative importance of those variables are not a matter of model structure. Analysis of PASs using an entirely different method (hierarchical partitioning) identified the same significant predictors (tables S2 and S3), with significant independent contributions of *SST* trend (*z*-score = 6.59) and average *SST* (*z*-score = 1.85) for the occurrence of negative PASs, while positive PASs were mostly influenced by *ER* trend (*z*-score = 6.91), age at maturity (*z*-score = 4.17), and, to a lesser extent, average *ER* and *SST* trend (*z*-score = 2.66 and 2.65, respectively).

### PAS and stock collapse

**Fig. 4.**
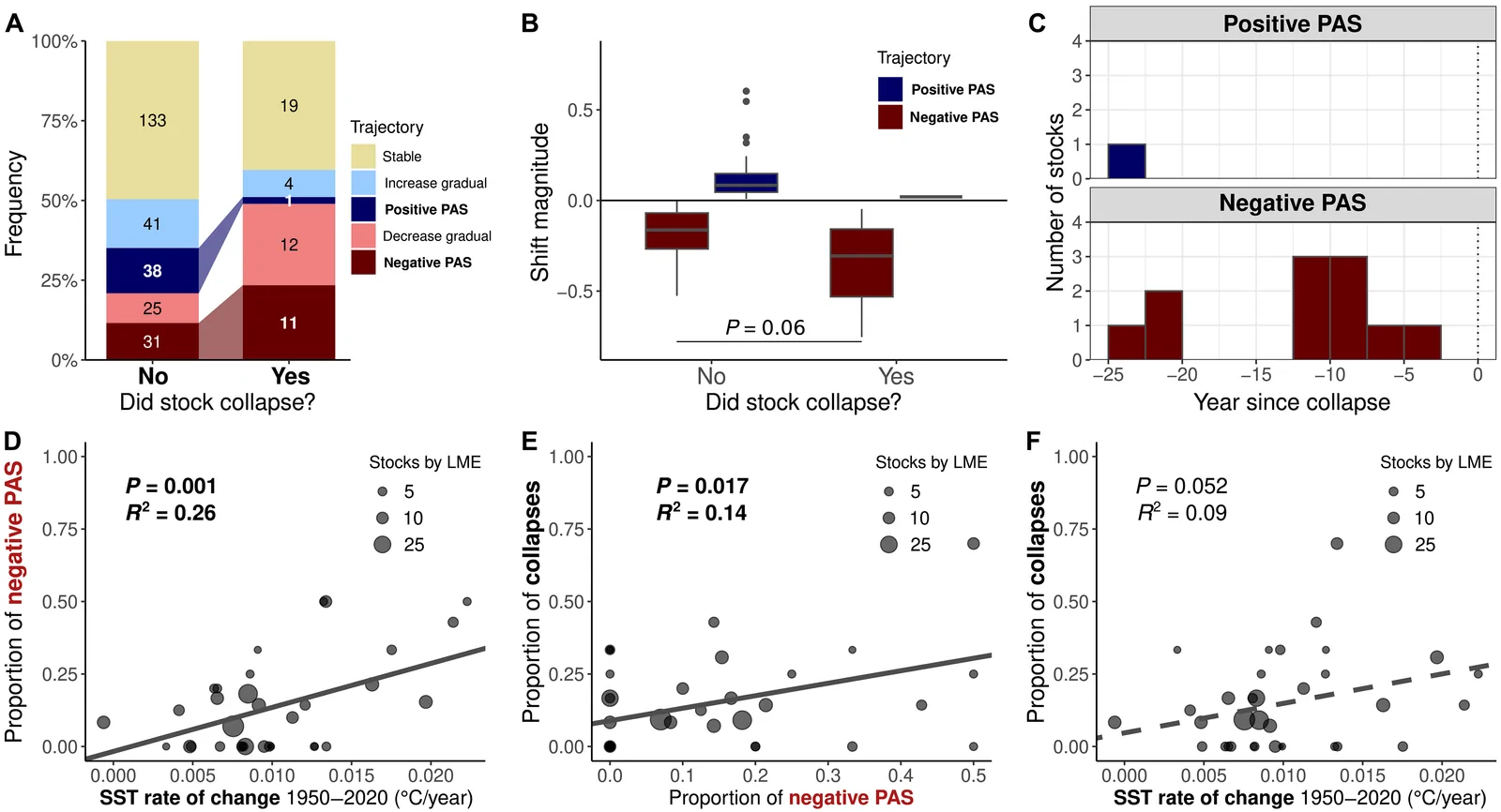
Relationships between PAS and stock collapse. The relationships are displayed at stock level [(A) to (C)] and at the scale of LMEs considering ocean warming rate [(D) to (F)]. (**A**) Proportion of trajectories depending on whether stocks ever reached collapsed state (defined as 25% of the mean biomass) with actual number of stocks indicated. (**B**) Standardized PAS magnitude for negative (dark red) and positive (dark blue) shifts and collapsed state. Student’s *t* test was performed on both groups. (**C**) Distribution of PAS location relative to the first year as collapsed (vertical dotted line), with positive PAS (top in blue) and negative PAS (bottom in blue). (**D** to **F**) Pairwise relationships between the proportion within LMEs of negative PAS, stock collapse, and *SST* rate of change between 1950 and 2020. Each circle represents one LME, whose size corresponds to the number of stocks. LMEs with less than two stocks were not included. Linear regression *P* value and adjusted *R*^2^ are displayed. Significant regressions (α = 0.05) are drawn with a solid line and dashed line otherwise.

Last, we tested the extent to which negative PASs were associated with stock collapse defined as being below 25% of the average stock biomass recorded to date. While not all negative PASs led to stock collapse (only 11 of 42 stocks, 26%, had negative PAS followed by stock collapse; Fig. 4A), among the stocks that did collapse, we found a higher proportion of negative PASs (23%; Fig. 4A) compared to those that did not collapse (12%; Fig. 4A). Collapsed stocks also tended to have more decreasing and fewer increasing productivity trajectories (Fig. 4A). On average, collapsed stocks experienced a stronger magnitude of negative PAS than stocks that did not collapse (*t* test, *P* = 0.06; Fig. 4B). Most of the negative PASs occurred between 4 and 12 years before the stock collapsed, while no such temporal lags were found for positive PASs (Fig. 4C).

The influence of *SST* on PAS and collapse was further investigated at the scale of LMEs. We evaluated the relationship between warming rate (*SST* change) between 1950 and 2020 and the proportion of stocks that underwent a PAS or a collapse. We found a significant positive linear relationship between negative PAS and warming rate (*P* = 0.001; Fig. 4D), meaning that LMEs with the most rapid warming rate were also those with the highest proportion of stocks that underwent negative PAS. The proportion of negative PAS is also positively correlated with the proportion of collapsed stocks (*P* = 0.017; Fig. 4E). However, we only found a marginally significant positive relationship between warming rate and the proportion of collapsed stocks (*P* = 0.052; Fig. 4F), even when using different definitions of collapse (0.05 < *P* < 0.48; fig. S4).

## DISCUSSION

In this work, we distinguished PASs in fisheries time series from gradual productivity trajectories using a systematic classification of trajectory types based on shape and trend (no change, linear, quadratic, and abrupt). We found that PASs were detected in more than 25% of stocks worldwide and that PAS occurrence varied in space and time. We provide evidence that large negative PAS frequently preceded stock collapses and were associated with a higher warming rate. Those findings could have critical implications for fisheries management in warming oceans.

Our results expand on the well-documented examples of regime shifts [e.g., (*43*, *44*)], giving a more complete picture of the prevalence of such shifts. Our classification aptly identifies stocks like Newfoundland cod (*23*), Baltic sea cod (*43*), or Japanese sardine (*45*), which underwent among the most prominent and rapid abrupt collapses previously characterized (full list available in table S1). We also identify others that were unexpectedly not extensively treated in the literature (e.g., Greenland halibut off Labrador Shelf–Grand Banks in the 1990s). Assuming that retrospective analyses of productivity trajectories can give good insights into how stocks are likely to react in the future, our approach enables the identification of stocks that could be more prone to abrupt decline and could be integrated in stock climate vulnerability assessments (*46*).

The prevalence of abrupt shifts we found (25%) is below those from previous analyses [46% (*47*) and 39% (*27*)]. The differences in prevalence could be explained by the set of stocks analyzed, the different relationships considered [e.g., stock recruitment (*47*)], the different models used [e.g., productivity-abundance relationship (*27*)], the use of alternative definitions of shift (*32*), and perhaps, most importantly, because our classification involved the congruence of two independent models to confirm a trajectory as abrupt, making the attribution of abruptness not only more strict but also probably more conservative. To avoid overfitting and focus on the most prominent shifts, a maximum of one abrupt shift per stock was characterized, while for long time series, several shifts may occur. This choice can also contribute to the underestimation of the prevalence of abrupt shifts, but the analysis of multiple shifts could also be conducted within the same framework. Our trajectory classification without the confirmation step doubles the prevalence of abrupt shifts, but the conclusions in terms of drivers identified and relationship between PAS and collapsed stocks remain largely the same (see the “Sensitivity analyses” section in Materials and Methods).

Among the life history, climate change, and fishing-related factors that have been proposed to affect stock collapses either regionally or globally, we found that the trend in *SST* was the variable explaining best the occurrence of negative PAS. The models only explained a limited fraction of the deviance, which suggests that the occurrence of abrupt shifts cannot be completely understood and predicted by the variables we considered. More specific drivers and context-dependent effects may be involved that hinder the identification of universal drivers of abrupt shifts. Yet, climate change, alone or in conjunction with other factors, has previously been identified as one of the most prominent drivers of marine regime shifts (*48*) and stock collapses (*39*). The LMEs that underwent the largest *SST* increases between 1982 and 2006 [>1°C (*49*)] are also those for which we found the largest proportions of stocks with negative PAS during the same time period, namely, the Baltic and North Sea, the East China Sea and Sea of Japan, and Newfoundland–Labrador Shelf. Fishing intensity was considered as the ratio of catch to biomass, and both variables were also used to estimate stock productivity. Caution is required when using the same model outputs to derive several parameters, as it could introduce falsely independent variables (*50*). Despite this concern, fishing intensity alone or in interaction with temperature did not explain negative PAS in our analyses, although an interaction between those drivers has previously been suggested for global (*39*) and regional (*51*) stock collapses. However, we found that the increase in fishing intensity was associated with positive PAS. This effect might correspond to the early stages of a fishery when the productivity of unfished stocks is usually low because the stock is assumed to be near its carrying capacity. This is in line with the basic principle of maximum sustainable yield, that productivity is maximized for intermediate levels of fish abundance corresponding to fishing mortality rates near those associated with maximum sustainable yield [*F*_MSY_ (*28*)]. No sign of abrupt recovery after collapse was found in the data, except for herrings in the North Sea, which recovered rapidly following reduction in fishing pressure—a result already well documented (*52*).

We accounted for life history essentially through maturity-related metrics and principal habitat, but in contrast with previous studies (*38*, *39*), we found no significant effect of those life history traits on negative shifts. Only the age at maturity, which is negatively correlated with a somatic growth rate, tended to be positively associated with positive shifts, meaning that rapid productivity increases were more often found in slower growing, later maturing species. This pattern is consistent with the periodic life history strategy (*53*) where delayed maturation and consequent large numbers of eggs can result in occasional very large recruitment events when these eggs encounter favorable conditions for early life survival. These periodic strategists often exhibit years of minimal or even negative productivity punctuated by occasional large year classes that create a period of high productivity as the year class ages and grows.

**Fig. 5.**
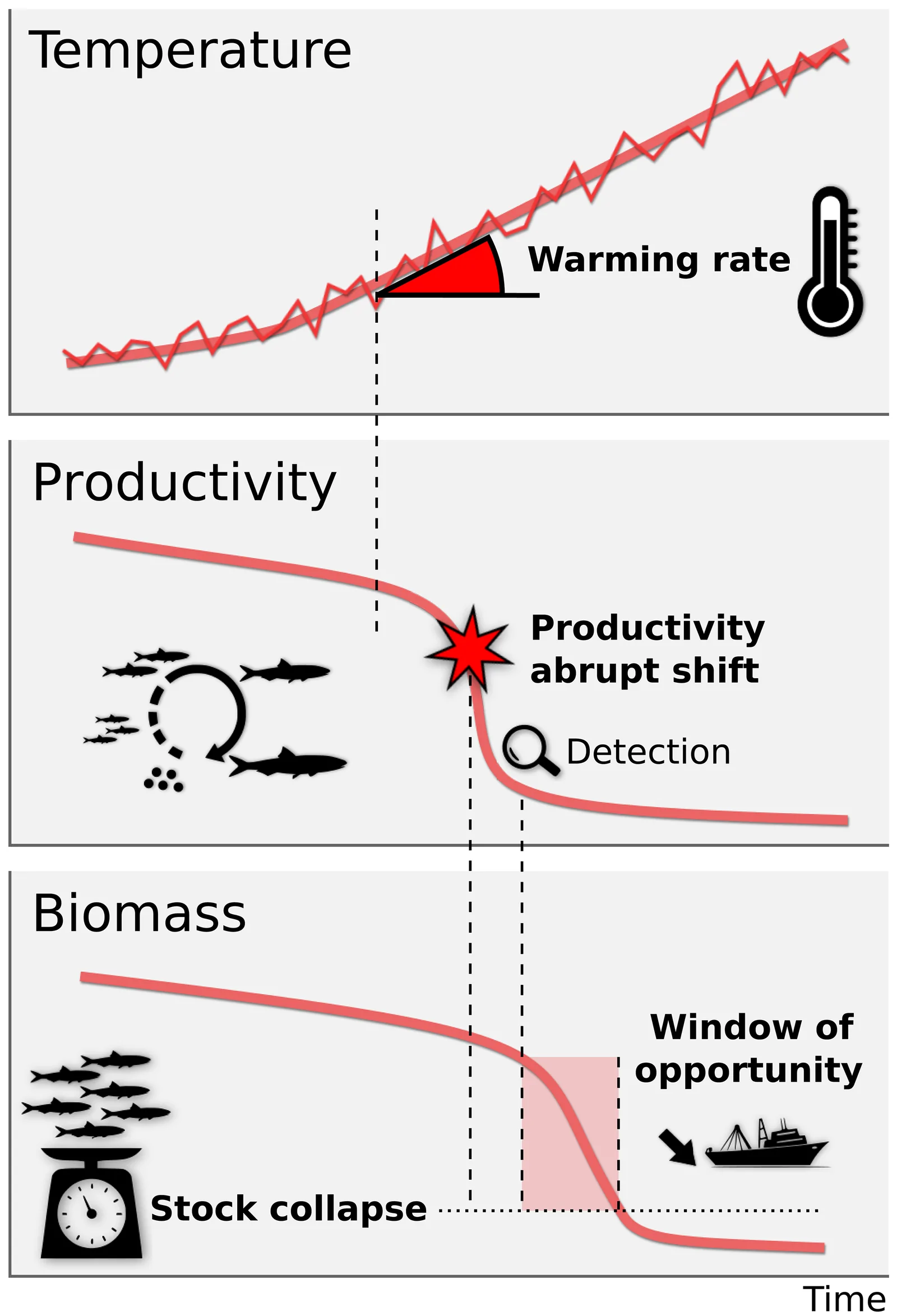
Proposed sequence linking warming rate to stock collapse through PASs. High warming rate might entail disruptions in stock recruitment, leading to a PAS and eventually a drop in biomass. Adjustment of management could be implemented within a time window starting with the detection of a productivity shift and ending with complete stock collapse.

We found that many negative PASs preceded stock collapse by several years, suggesting that within the proposed sequence of events starting from temperature rise, to productivity shift, and stock collapse (Fig. 5), there is a critical time window for fishery managers to take action to avoid collapse, although the quantification of the shift detection delay in practical conditions has yet to be estimated. Rapid reductions in fishing pressure have been shown to be the key factor in determining recovery rates of collapsed stocks (*54*) and may also serve as a short-term measure, in addition to other multilevel and climate-resilient actions (*5*, *55*), to prevent collapse if negative PASs can be detected quickly enough. The key elements here are likely to be methods for rapid detection of PAS (*56*), frequent stock assessment updates with minimal lags between data collection and assessment, and mechanisms—such as harvest control rules—that quickly translate negative PAS into reduced fishing pressure (*57*). Our results also suggest that gradual stock collapses were associated with lower levels of warming, while areas that underwent rapid warming were more likely to experience negative abrupt shifts. There is evidence for such warming-driven declines notably in the case of insufficient management adaptation (*35*), but a more extensive coverage of stocks with lower warming rate would be necessary to confirm this. All these results are robust to different definitions of stock collapse (see the “Sensitivity analyses” section in Materials and Methods).

The abrupt shifts in productivity that we identified here are different from marine ecosystem regime shifts. Regime shifts frequently involve long-lasting community-wide changes in multiple components of the food web, often precipitated by broader changes in ocean climate. For example, the regime shifts identified in the Bering Sea and eastern North Pacific Ocean included changes in ocean circulation, lower trophic level productivity, and changes in community composition and abundance of marine invertebrates, fishes, and mammals (*58*–*60*). Here, we did not link the PASs we detected at the population level to broader ecosystem changes although it is plausible that they are not isolated events specific to the dynamics of these species (*12*, *16*). It would be worth exploring whether the PASs we found are either driving community-wide shifts or are triggered by large-scale ecosystem shifts.

While the RAM Legacy database has broad geographic coverage, it does not reflect the actual distribution of fisheries worldwide and is biased toward intensively monitored stocks mostly from wealthy nations at temperate latitudes (*61*). Because of various reasons ranging from unreported fishing activities (*62*) to the lack of local dedicated agencies, fisheries from tropical latitudes are more often data limited or monitored for a lower amount of time, which limits reliable shift detection. Yet, conspicuous examples of regime shifts have been reported in tropical areas like in coral reef communities (*63*) and should not be ignored. The correlation we found between a warming rate and negative PAS suggests that marine areas like the North-West and South Atlantic or South Indian Oceans that are projected to be hotspots of rapid temperature increase in the next decades (*64*) require careful attention and monitoring. This is a challenging task since it has been suggested that regional fisheries agencies are still struggling to account effectively for climate change in their practices (*65*).

The fact that ocean warming is increasingly altering stock productivity patterns, often before stock collapses actually occur, implies that the collapse-based reference points used in fisheries management might rapidly prove insufficient. Our findings suggest that incorporating PASs as warning signals to stock biomass collapses into harvest control rules could enable earlier and more adaptive responses. This could pave the way for advancing more productivity-based management practices in which the monitoring and even the anticipation of productivity shifts (*56*) could enhance sustainability.

## MATERIALS AND METHODS

### Fisheries data

Time series of catch and stock biomass were downloaded from the freely available RAM Legacy Stock Assessment database [RAMLDB v4.61 (*61*)]. From those, we estimated stock productivity (or surplus production) time series, which is a biological variable particularly relevant to investigate putative regime shifts in fisheries (*27*). Abrupt shifts in productivity time series have been previously used as a hallmark of regime shift for some fish stocks (*43*, *44*). Surplus production can be assimilated to stock productivity if it is independent of biomass, which has been suggested to be the case for most of the stocks (*27*). We therefore refer to surplus production as productivity. The productivity *S*(*t*) in the year *t* was estimated from catch and total biomass time series according to the following formula

S(t)=B(t+1)−B(t)+C(t)

with *B*(*t*) stock total biomass and *C*(*t*) catch in the year *t*. While catch values are raw data, often assumed to be measured without error, biomass estimates are model outputs, and concerns have been raised when considering model outputs as input data (*50*). Here, the greatest concern is that interannual smoothing of biomass time series by the assessment model could cause us to miss true abrupt shifts. This concern is minimized by the fact that most biomass time series in the database are from highly flexible statistical catch at age models that allow for substantial process error [e.g., interannual variation in reproductive output or recruitment (*61*, *66*)]. Our data filtering criteria (described below) removed a small number of stocks where smooth biomass trajectories were clearly a model artifact.

The RAMLDB includes stock assessments from several phyla including mollusks, crustaceans, and vertebrates (fishes). For consistency, we only focused on marine fish stocks including both ray-finned and cartilaginous fishes but excluding anadromous fishes of the family Salmonidae and ignored data before 1950 that sometimes corresponded to model extrapolations. Fish productivity time series with no missing data points were estimated for 397 stocks, but a total of 82 were discarded either because they had time series length below 25 years (68 stocks) or because biomass estimates were apparently generated from deterministic models which could only produce smooth changes (14 stocks). The classification was thus performed on 315 stocks corresponding to 161 taxa, among which 158 were at the species and 3 at the genus level. Median time series length was 41 years, and the longest time series covered 71 years. To allow for interstock comparisons, productivity was normalized by average stock biomass following (*36*).

### Time series classification

Productivity time series were fitted with four different types of model—intercept-only (no change), linear trend, quadratic trend, and abrupt change—and Akaike Information Criterion corrected (AICc) for small sample size were computed for each model fit. The best trajectory was considered to be the model with lowest AICc and validated following (*67*). To validate abrupt trajectories, an independent breakpoint detection method [asdetect (*68*)] was run, and we checked whether both methods agreed on the shift date with a tolerance of 5 years. For quadratic and linear cases, we tested the significance of the higher-order coefficient. We used the classification with default parameters for the asdetect method (anomalous rate of change equal to three medians absolute deviations and detection threshold set to 0.15) that have been tested to be most reliable for time series of at least 25 time points (*67*). To avoid uncertain shifts, shifts less than 5 years from the start or the end of the time series were not considered. If the abrupt shift model and the asdetect method agreed that an abrupt shift occurred but disagreed on the year of the shift, the year identified by the abrupt shift model was used. We also computed three classification quality score to assess different aspects of model reliability, namely, the relative support for each fitted model (AICc weight), model choice robustness to individual data point removal (leave-one-out score), and the ratio between variation not explained by the model and the overall variation (normalized RMSE). This framework permits the identification of at most one shift in each time series, which is assumed to be the largest one if several might be present. The standardization by stock average biomass changes the absolute AICc values but does not affect model ranking or best model choice.

### Spatial data

A stock represents a consistent population unit that is spatially bounded. Most stock polygons were extracted from an existing dataset (*37*). An addition of 64 stock polygons to the dataset was made following the same methodology either by combining fishing subareas or divisions, approximating polygons to already existing ones, or digitizing fishing areas based on individual assessment data. All added polygons were processed using QGIS v3.16. Stocks were grouped by FAO Major Fishing Areas as directly available from the RAMLDB dataset. We also grouped stocks by LMEs that represent biogeographically more relevant units. We assigned each stock to the LME with which stock polygons overlapped most, except for high seas fisheries for which they were assigned to main ocean areas.

### Explanatory variables

Potential explanatory variables were gathered for each taxa or stock, spanning life history, environmental, and fishing-related pressure. To avoid missing data, we used imputed life history traits from the FishLife database (*69*) and selected two relevant variables among the least correlated ones, namely, age and length at maturity (fig. S5A). Primary habitat was retrieved from FishBase (*70*) with classes grouped as pelagic (pelagic-neritic, pelagic-oceanic, and bathypelagic) and demersal (bathydemersal, benthopelagic, demersal, and reef-associated). *SST* was used as the most relevant environmental variable using data from the Met Office HadISST1 dataset (*71*) from 1870 to 2020 on a monthly basis (then averaged annually) and with one degree spatial resolution. Annual *SST* maps were clipped on the basis of stock or LME boundaries and averaged to obtain a single *SST* time series per stock (*37*). *ER* was used as a proxy for fishing intensity and corresponds to the ratio of catch to total biomass in the same year. Although derived from the same time series of catch and biomass, we found no noticeable relationship between surplus production and *ER* for most of the stocks retained for the analyses, which alleviates the concerns of using a few model outputs to estimate several parameters (*50*). *ER* relative to that at maximum sustainable yield *U/U*_msy_ may have been a better proxy since it accounts for intrinsic differences in productivity among stocks but was not available for 15% of the stocks. *SST* and *ER* averages and trends were estimated from the start of productivity time series up to the shift if any was detected or to the latest year available otherwise. A classification of *SST* time series in abrupt, quadratic, linear, and no change trajectory types suggested that abrupt changes were not common (<10%), which supports the use of linear trends as appropriate to describe changes in *SST*. The absence of strong multicollinearity of explanatory variables was determined by performing pairwise Pearson correlation tests (fig. S5B).

To test the association between stock collapse and PAS, we defined a stock as collapsed if biomass in a given year was below 25% of the average stock biomass recorded to date as previously defined in the literature (*36*) and if such threshold was crossed for at least 2 consecutive years to limit artifacts. We focused on whether a stock ever collapsed and the first year of collapse. We repeated the analyses that involved collapsed status with alternative frequent definitions from the literature (*32*), namely, 10% of maximum biomass and 15 and 50% of average biomass. The correlation between PAS, stock collapse, and *SST* rate of change was also evaluated in terms of proportions at the scale of LMEs and fitted using weighted least squares with the number of stocks available per LME as weight to limit the influence of LMEs with few stocks.

### Statistical analyses

We assessed the homogeneity of trajectories found across regions and taxonomic orders separately using chi-square test and computed *P* values by Monte Carlo simulation using 10^5^ replicates to deal with cases of low expected values. We used logistic GAMMs to assess the effect of independent variables on the occurrence of PASs using the mgcv package (*72*). Positive and negative shifts were considered separately as dependent variables against all other trajectories to contrast conditions of abrupt shifts against all conditions available. The effects of life history, environmental conditions, and fishing intensity were estimated as fixed effects by considering age at maturity, length at maturity, main habitat, average and trend in *SST*, and average and trend in *ER* altogether. We also included the interaction between average *SST* and *ER* and trends in *SST* and *ER* to investigate potential synergistic effects. Numerical variables were standardized to allow comparison of odd ratios across predictor variables. To account for the spatial dependence between observations, we included stock centroid coordinates as smooth terms following a common practice in such models with the default thin plate regression spline as smoothing basis. Taxonomic orders and families were treated as a random effect to account for broad phylogenetic relationships, and time series length that can influence shift detection was also included as a random effect. Two stocks from the Japanese Seto inland sea lacked *SST* data due to limited spatial resolution and were thus discarded for these analyses.

An alternative approach was also performed to assess the contribution of each predictor variable independently and in conjunction with the other predictors on the occurrence of abrupt shifts. This approach controls for the possible influence of unchecked multicollinearity among variables. We used the hier.part package (*73*) to run hierarchical partitioning and randomization tests with 999 repetitions on positive and negative shift occurrence as binomial variables with a logit link function. Phylogeny was not used for these analyses because of excessive number of groups in each taxonomic level.

### Sensitivity analyses

We also ran the classification without the step for abrupt shift confirmation to determine how frequently this step was eliminating potential abrupt shift classifications. Without the confirmation step, the proportion of stocks classified as PAS reached 51.4% with similar proportions of negative (26%) and positive (25.4%) shifts (fig. S6). We also repeated the GAMM analysis of predictors of PAS with this less strict classification for PAS. In that case, we found a marginal effect on negative PAS for *SST* change (*odds ratio* = 1.19, *CI*_*95%*_ = [0.91, 1.58], *P* = 0.189; fig. S7A), average *ER* (*odds ratio* = 1.37, *CI*_*95%*_ = [0.96, 1.96], *P* = 0.080; fig. S7A), and age at maturity (*odds ratio* = 1.45, *CI*_*95%*_ = [0.88, 2.41], *P* = 0.142; fig. S7A). For positive PAS, we found an effect of average *ER* (*odds ratio* = 0.44, *CI*_*95%*_ = [0.25, 0.77], *P* = 0.004; fig. S7B), *ER* trend (*odds ratio* = 1.90, *CI*_*95%*_ = [1.09, 3.31], *P* = 0.023; fig. S7B), and marginally of the interaction between average *SST* and *ER* (*odds ratio* = 0.63, *CI*_*95%*_ = [0.39, 1.03], *P* = 0.066; fig. S7B).

We repeated the GAMM with fishing intensity relative to that at maximum sustainable yield (*U/U*_msy_) instead of *ER*. *U/U*_msy_ accounts for intrinsic species differences in withstanding fishing pressure but was available only for 269 stocks. Similar effects to the initial models in Fig. 3 were found in estimates although not significant for *SST* change alone but significant for the interaction between *SST* and *ER* trends (*odds ratio* = 2.55, *CI*_*95%*_ = [1.37, 4.74], *P* = 0.003; fig. S7C) with, in addition, a significant positive effect of *U/U*_msy_ trend (*odds ratio* = 2.93, *CI*_*95%*_ = [1.59, 5.42], *P* < 0.001; fig. S7C). The differences with the initial model using *ER* could be due to the loss of one-third (14 of 42) of the stocks with negative PAS that lacked *U/U*_msy_, among which 6 of 11 that also collapsed. The stocks missing *U/U*_msy_ also tended to have a higher warming rate. The results of this sensitivity analysis might not be representative of the stocks included for all other analyses and thus should be interpreted with those limitations in mind. Similar effects to the initial models in Fig. 3 were also found for positive PAS, although not significant, with age at maturity (*odds ratio* = 1.61, *CI*_*95%*_ = [0.88, 2.94], *P* = 0.122; fig. S7D), average *U/U*_msy_ (*odds ratio* = 0.62, *CI*_*95%*_ = [0.35, 1.08], *P* = 0.091; fig. S7D), and the interaction between U/U_msy_ and *SST* (*odds ratio* = 0.64, *CI*_*95%*_ = [0.40, 1.03], *P* = 0.066; fig. S7D).

Without the step for abrupt shift confirmation, the proportion of collapsed stocks that also shifted negatively remained very close with 21 stocks of 82 (26%; fig. S8A) compared to 11 stocks of 42 (26%; Fig. 4A) with abrupt shift confirmation and using the same collapse definition. At the scale of LMEs, the relationship between the proportion of negative PAS and warming rate (*P* = 0.006; fig. S8D) and between the proportions of negative PAS and collapse stocks (*P* = 0.009; fig. S8E) remained positive and significant. We also tested the robustness of the results related to stock collapse by using different thresholds of collapse (10% of maximum stock biomass and 15 and 50% of the average stock biomass), which gave similar results in terms of relationship with negative PAS (fig. S9) and warming rate (fig. S4).
