# Supplementary material for: Abrupt declines in fish productivity often precede stock collapses in marine fisheries
Source: Sci Adv. 2026 Sep 2;12(36):eaed7911. doi: 10.1126/sciadv.aed7911 (PMC13537261; doi:10.1126/sciadv.aed7911)
Supplement: Supplementary file 1 [file sciadv.aed7911_sm.pdf]

Science Advances
AAAS

Supplementary Materials for

Abrupt declines in fish productivity often precede stock collapses in marine fisheries

Mathieu Pélissié *et al.*

Corresponding author: Mathieu Pélissié, mathieu.pelissie@ens-lyon.fr

Sci. Adv. **12**, eaed7911 (2026)
DOI: 10.1126/sciadv.aed7911

This PDF file includes:

Figs. S1 to S9
Tables S1 to S3

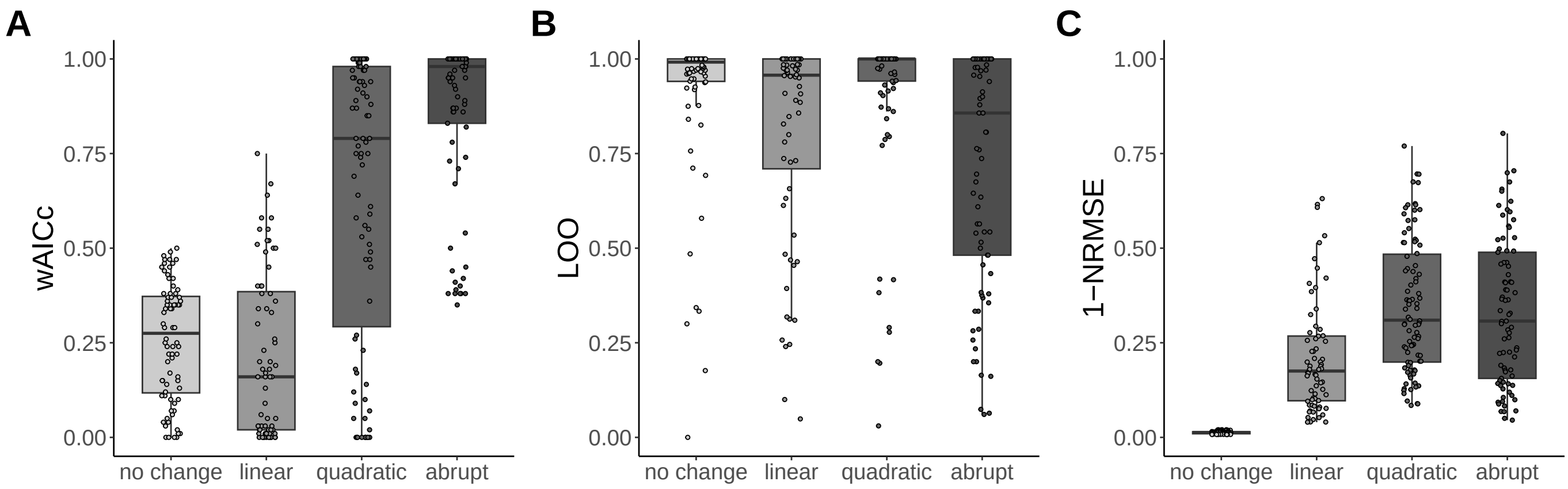

Figure S1: Distribution of classification quality scores by trajectory class. (**A**) wAICc expresses how much the best model performs in terms of AICc compared to the others. (**B**) LOO indicates how robust is the model choice to the removal of individual data points. (**C**) (1-NRMSE) gives the proportion of variation explained by the model. For time series classified as ”no change”, (1-NRMSE) values are expected to be close to zero since the best model does not differ from the mean of the time series. Values of wAICc below 0.25 correspond to time series classified as abrupt by the fitting method but not validated as abrupt by the *asdetect* method.

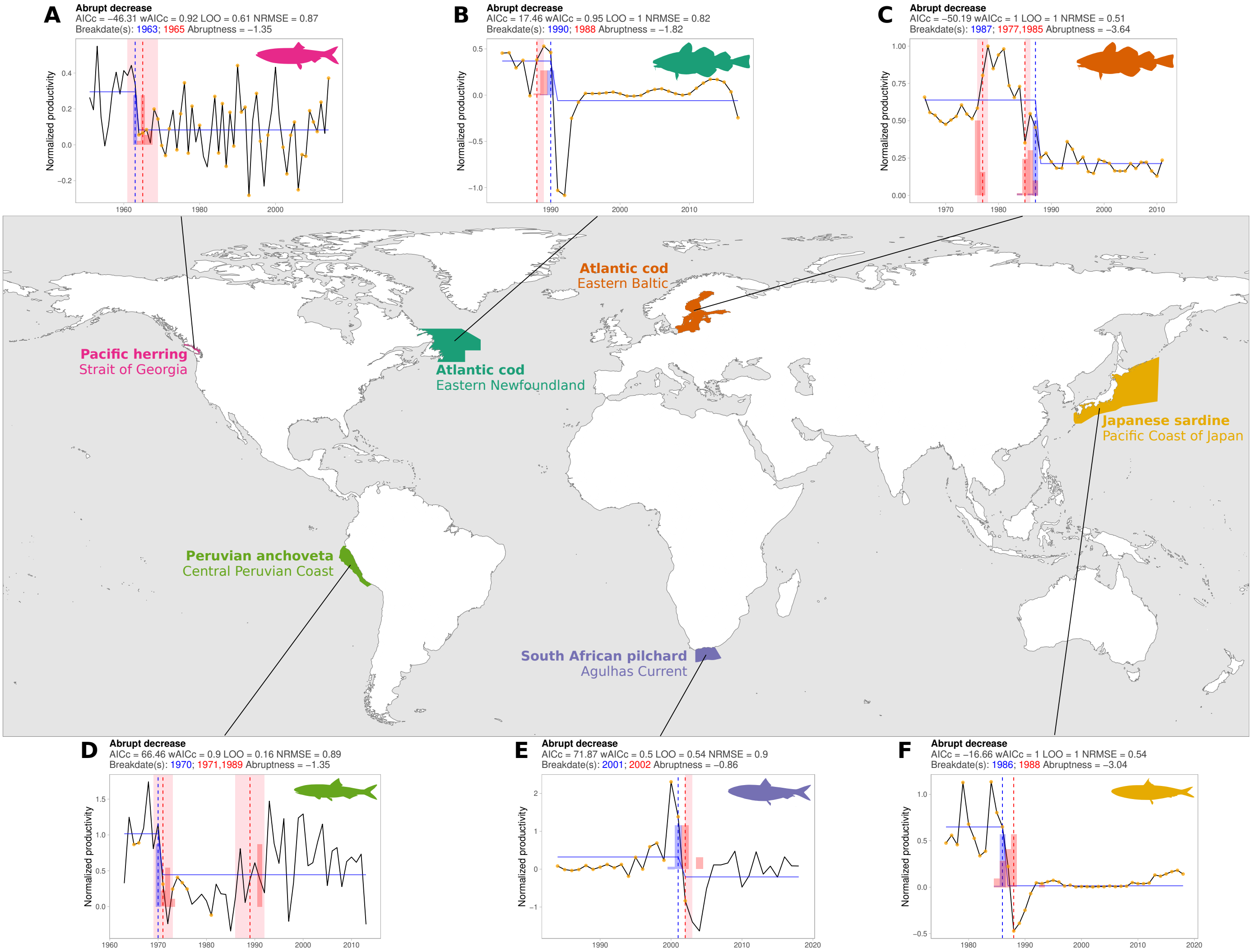

Figure S2: A selection of six stocks well documented for having experienced significant shifts, for which the classification correctly identified as negative PAS. The time series classification output is displayed along with the stock boundaries for Pacific herring from Strait of Georgia (HERRSOG, **A**), Atlantic cod from Southern Labrador - Eastern Newfoundland (COD2J3KL, **B**), Atlantic cod from Eastern Baltic sea (CODBA2532, **C**), Japanese sardine from the Pacific Coast of Japan (PILCHPJPN, **D**), South African pilchard from Aguilhas Current (SARDSAS, **E**), and Peruvian anchoveta from Central Peruvian Coast (PANCHPERUNC, **F**). Fish silhouettes were obtained from PhyloPic images (www.phylopic.org/) under CC0 1.0 or PDM 1.0 licenses.

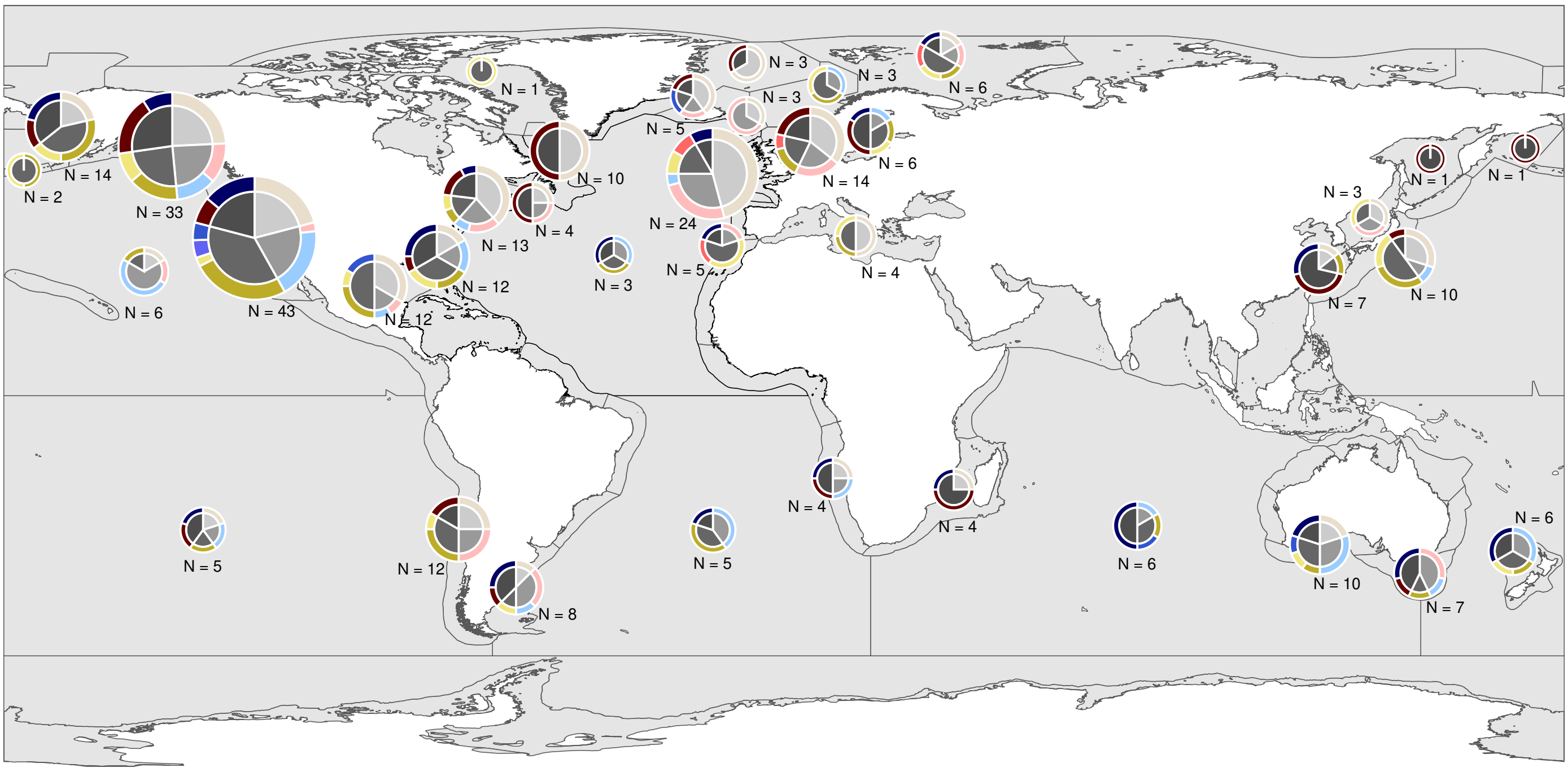

Figure S3: Spatial distribution of trajectories by large marine ecosystems (LMEs). The size of the charts is indicative of the number of stocks, which is also indicated.

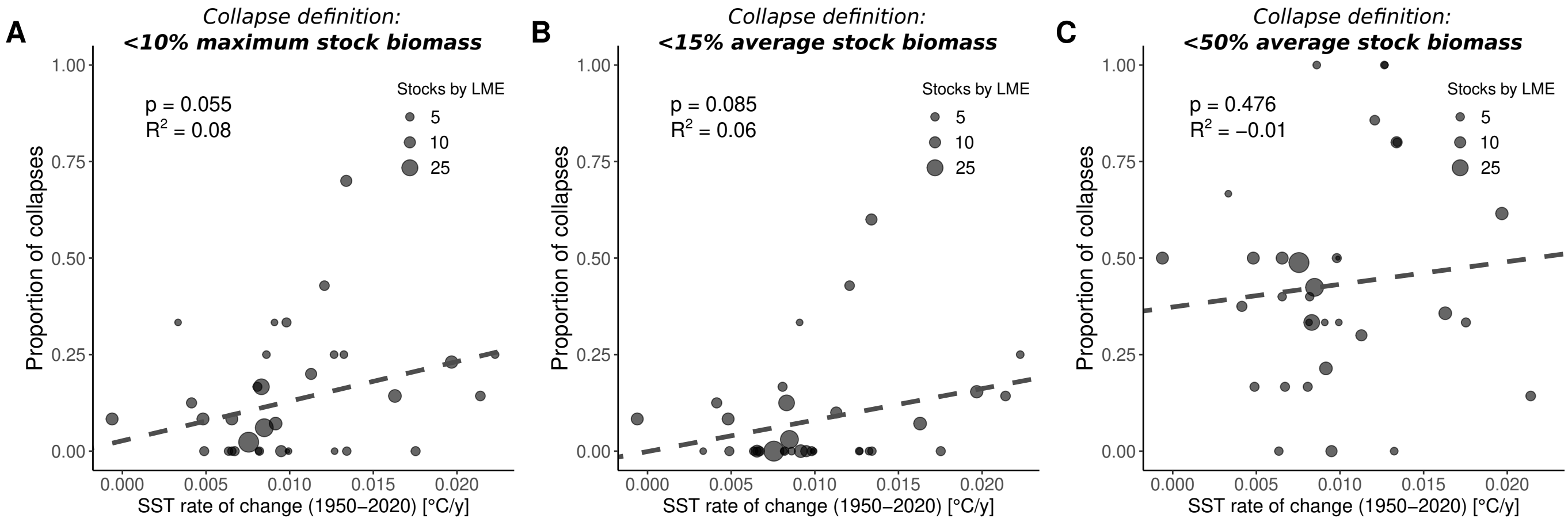

Figure S4: Relationship between the proportion of stock collapse and SST rate of change for different collapse definitions. Collapse is either defined as 10% of the maximum biomass (**A**), 15% (**B**), or 50% of average biomass (**C**). The values were computed at the level of LMEs between 1950 and 2020. Each circle represents one LME, whose size corresponds to the number of stocks. LMEs with less than two stocks were not included. Linear regression p-value and adjusted R-squared are displayed. Significant regressions are drawn with a solid line, and dashed line otherwise.

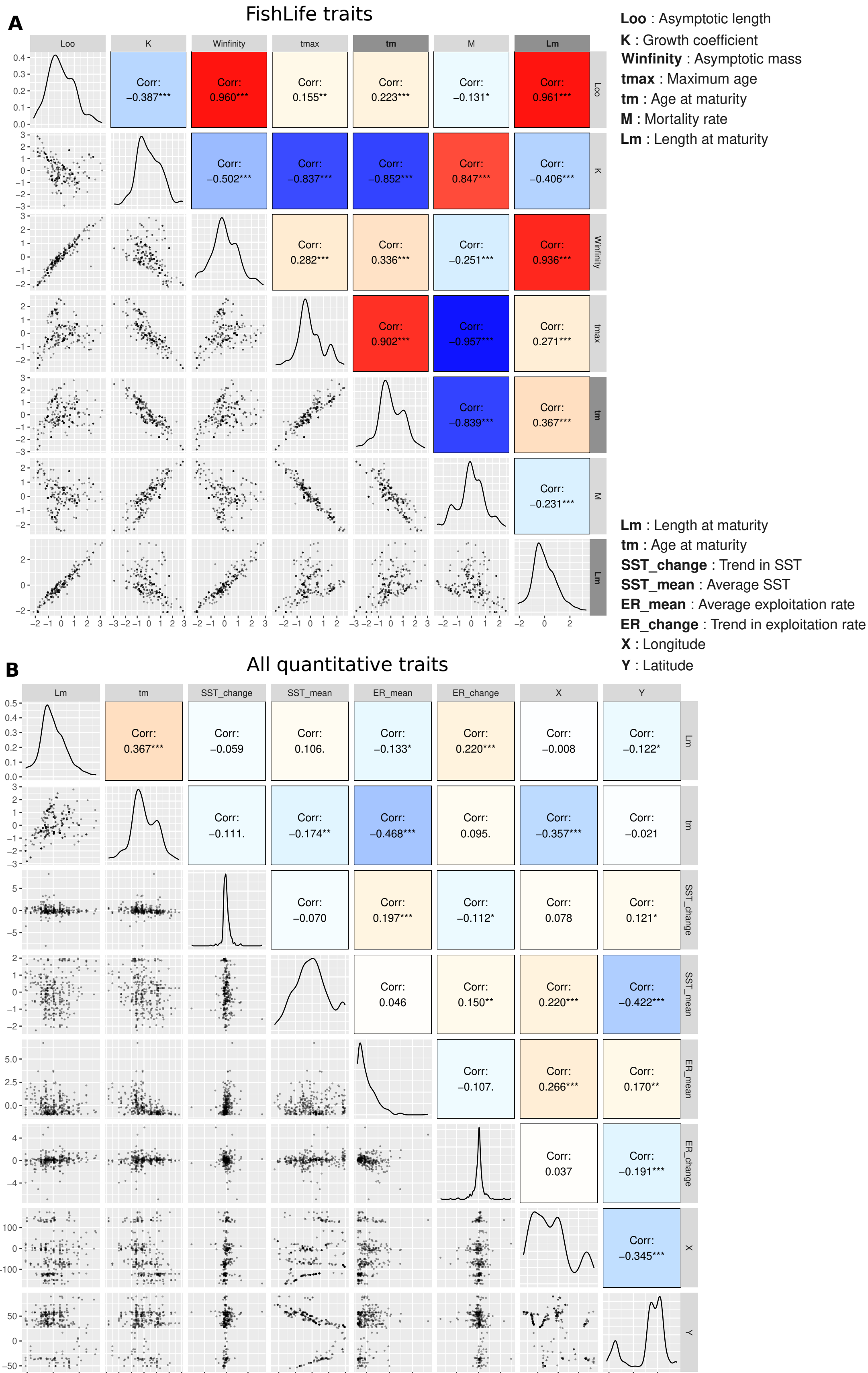

Figure S5: Correlation plots for trait variables of the available stocks. Pairwise Pearson correlation coefficient between explanatory variables, for FishLife traits (**A**) and for all quantitative traits used in the GAMMs (**B**). Background color in the upper triangle indicate level of correlation, lower triangle show correlation biplots, and density distribution are shown on the diagonal. Acceptable correlation level are below 0.7. All variables except stock centroid coordinates X and Y were scaled as used in models.

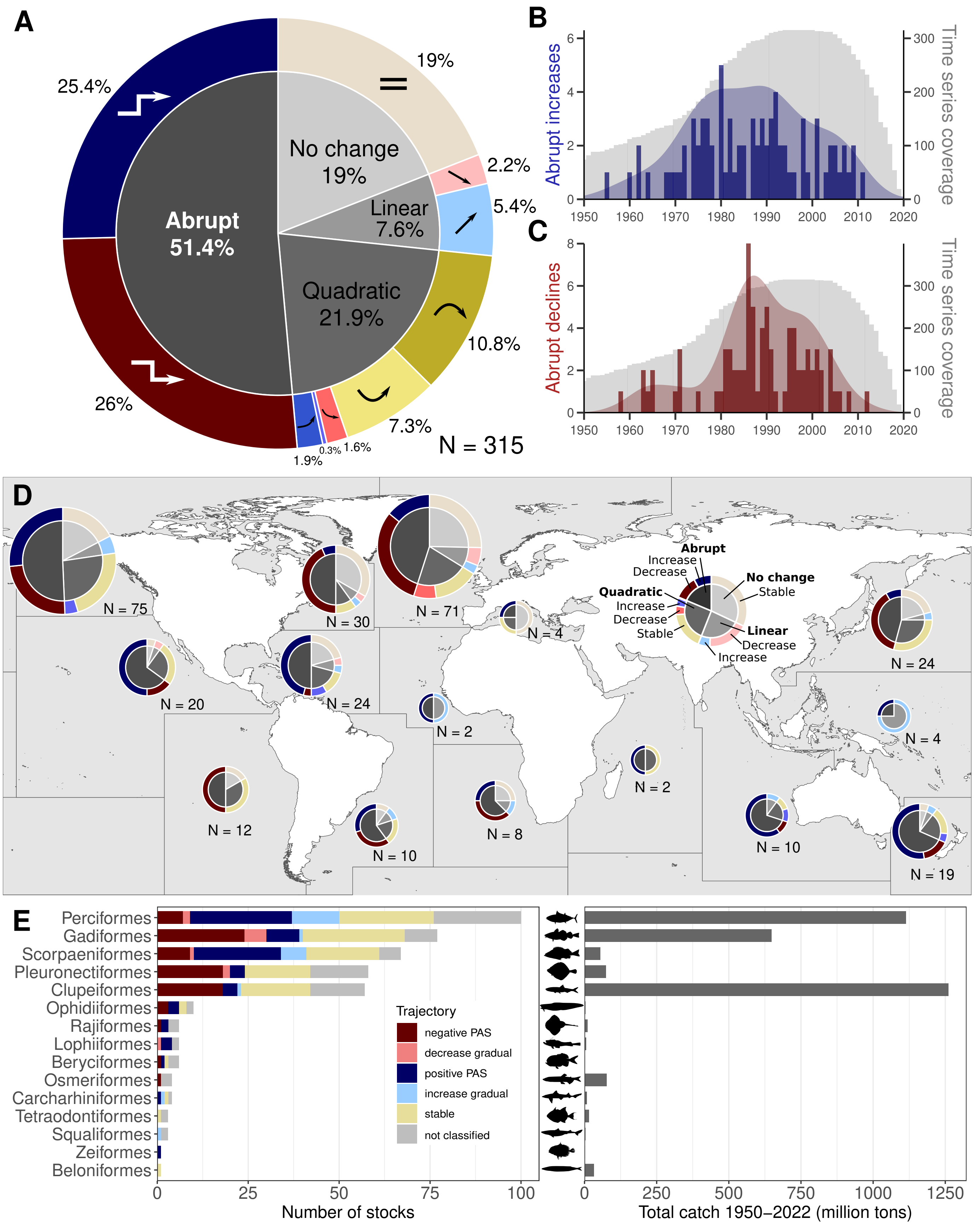

Figure S6: Distribution of productivity trajectory types in space, time, and taxonomic groups for trajectory classification without abrupt shift confirmation. Trajectory classification is based only on AICc comparison of four different models. (**A**) Overall proportion of all productivity trajectory types, with the inner pie chart indicating trajectory shape and the outer layer specifying the trend. (**B**-**C**) Temporal distribution of positive (**B**) and negative PAS (**C**) (color bars and density) with in gray the coverage (i.e., number of stocks with data available in each year) of the time series classified.(**D**) Spatial distribution of all trajectory types by FAO major fishing areas. The size of the charts is indicative of the number of stocks, which are also displayed. (**E**) Productivity trajectories by taxonomic order and total catch worldwide between 1950 and 2022 based on FAO data. Stocks that were not classified (e.g., length below 25 years) are in gray. Fish silhouettes were obtained from PhyloPic images (www.phylopic.org/) under CC0 1.0 or PDM 1.0 licenses.

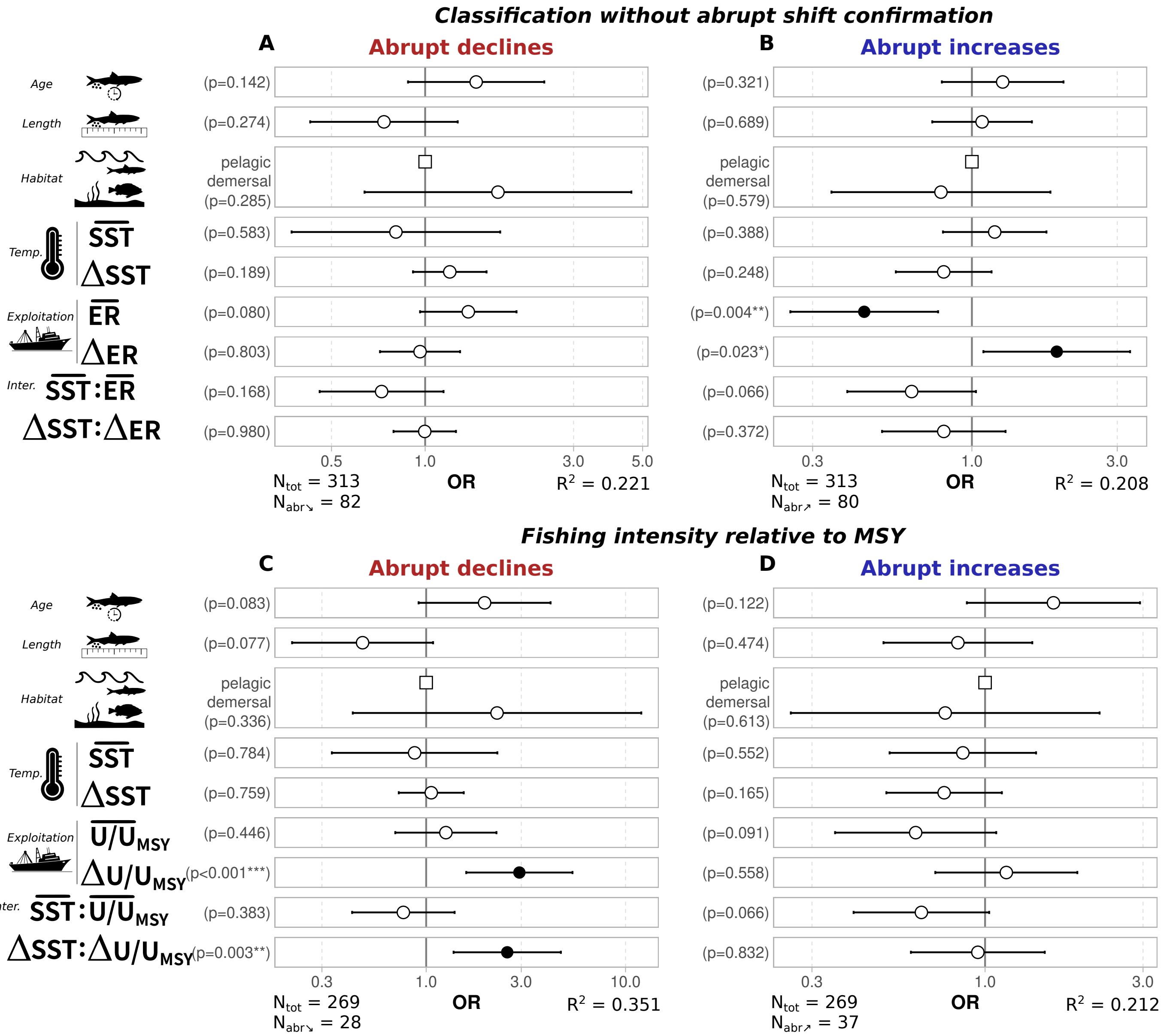

Figure S7: Quantification of drivers of PAS with alternative classification or fishing intensity variables. Generalized additive mixed model (GAMM) coefficient estimates for models accounting for negative (**A**, **C**) and positive (**B**, **D**) PAS against all other trajectories. (**A**, **B**) Classification used consists only in AICc comparison (no confirmation of abrupt shifts) with *ER* as proxy for fishing intensity. (**C**, **D**) Classification used is identical to that in the main text fig. 3 with average and trend in U/U_{MSY} instead of *ER*. See the caption of main text fig. 3 for more details.

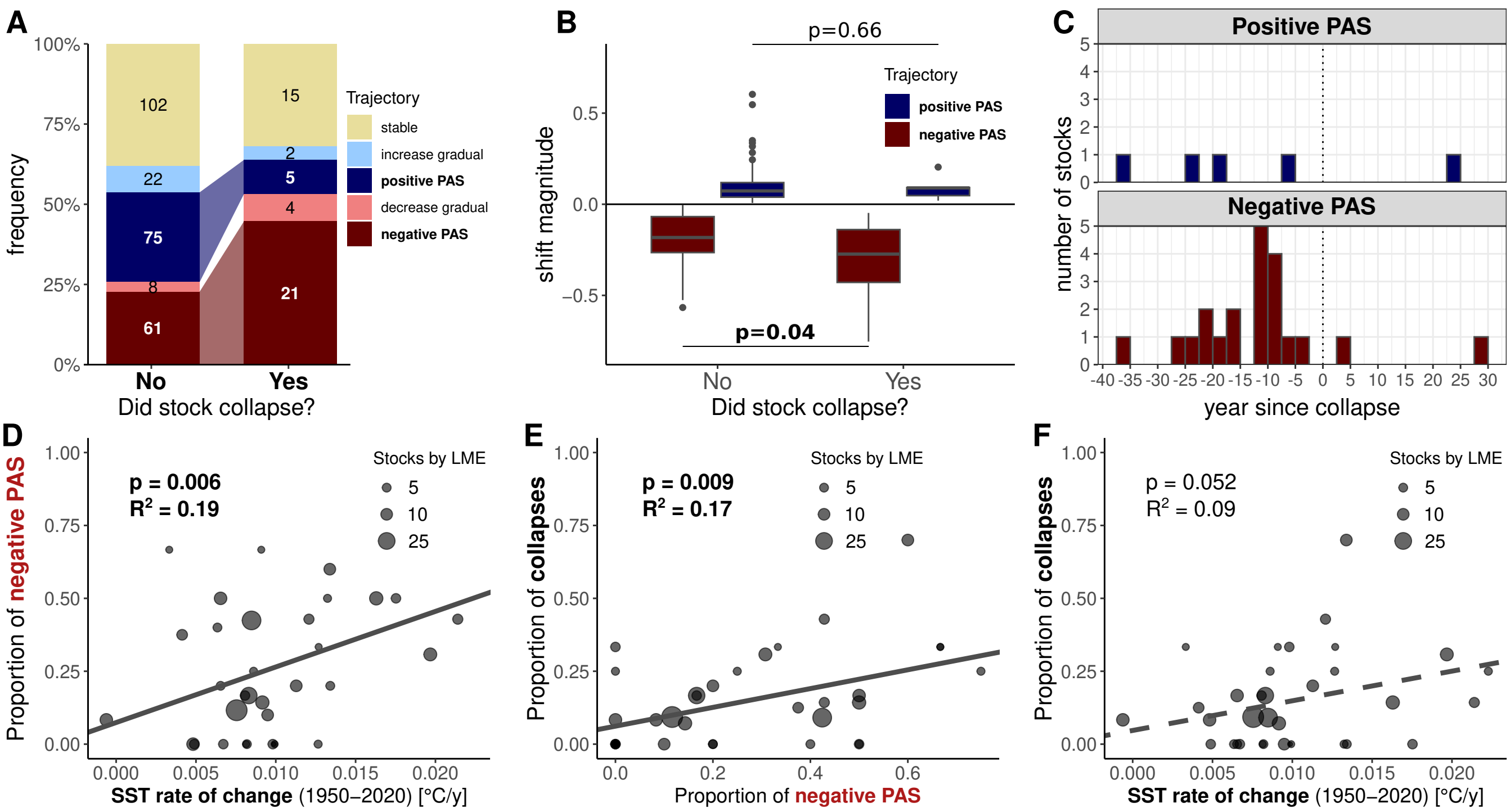

Figure S8: Relationship between PAS and stock collapse for trajectory classification without abrupt shift confirmation. The relationships are displayed at stock level (**A**-**C**) and at the scale of Large Marine Ecosystems (LMEs) considering ocean warming rate (**D**-**F**). Trajectory classification is based only on AICc comparison of four different models and collapse is defined as falling below 25% of the mean biomass. See the caption of main text Fig. 4 for more details.

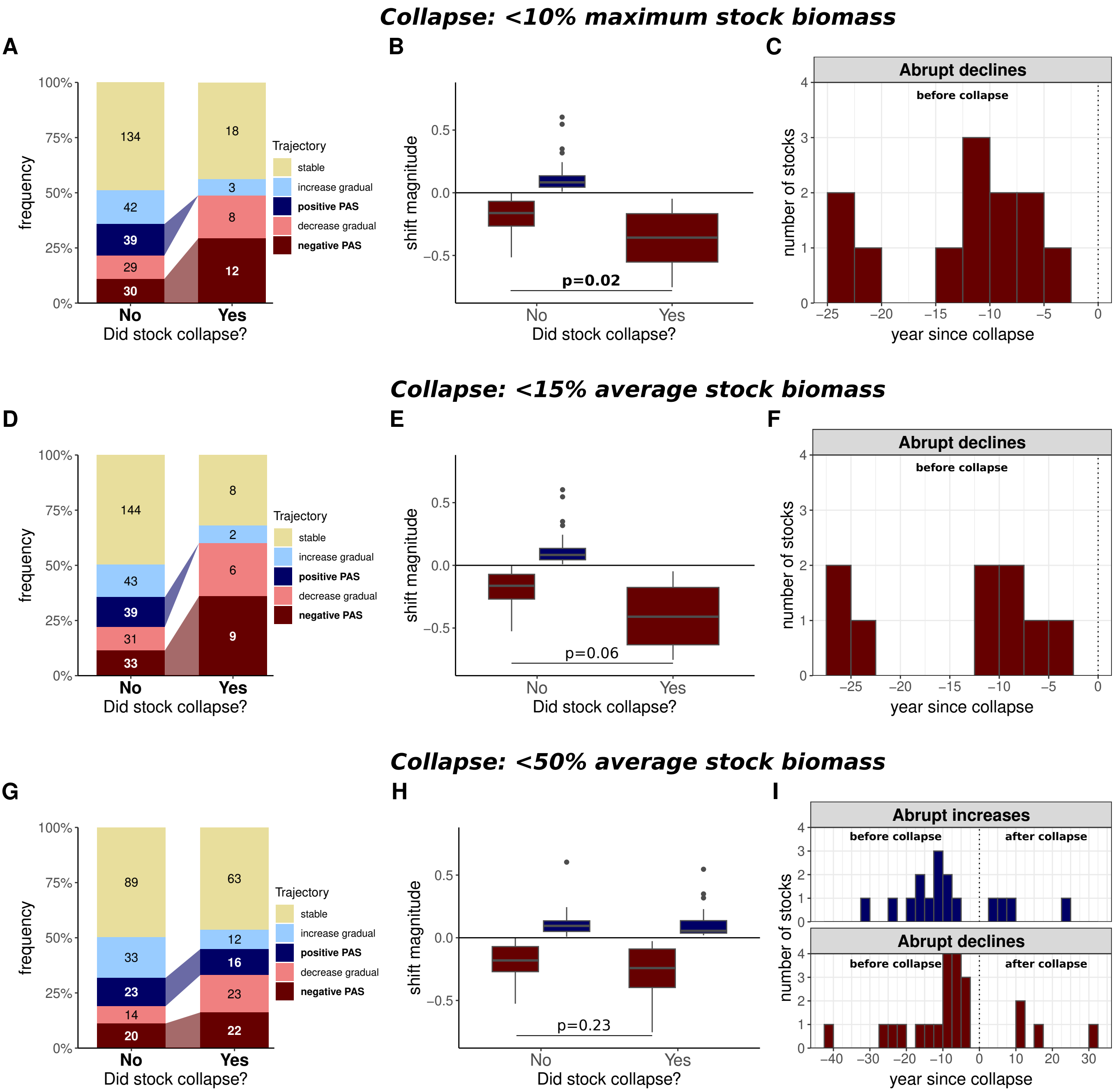

Figure S9: Relationship between PAS and stock collapse at stock level for different thresholds of collapse. Collapse is either defined as 10% of the maximum biomass (**A-C**), 15% (**D-F**), or 50% of average biomass (**G-I**). See the caption of main text Fig. 4 for more details.

Table S1: Fish stocks included in the analyses with the productivity trajectories and details of abrupt shifts (if detected) according to the full time series classification.

Stock name	Species	Large Marine Ecosystem	Class	Trajectory	Shift year	Shift magnitude
ACADRED2J3K	*Sebastes fasciatus*	Labrador - Newfoundland	no change	stable constant	NA	NA
ACADREDUT3	*Sebastes fasciatus*	Scotian Shelf	no change	stable constant	NA	NA
ACMACKSARG	*Scomber colias*	Patagonian Shelf	linear	increase constant	NA	NA
ALBAMED	*Thunnus alalunga*	Mediterranean Sea	no change	stable constant	NA	NA
ALBANATL	*Thunnus alalunga*	North Atlantic Ocean	quadratic	stable concave	NA	NA
ALPLAICBSAI	*Pleuronectes quadrituberculatus*	East Bering Sea	abrupt	decrease abrupt	1981	-0.06
ALSKABSAI	*Bathyraja parmifera*	East Bering Sea	abrupt	increase abrupt	1980	0.05
AMPL23K	*Hippoglossoides platessoides*	Labrador - Newfoundland	no change	stable constant	NA	NA
AMPL3LNO	*Hippoglossoides platessoides*	Labrador - Newfoundland	abrupt	decrease abrupt	1983	-0.18
ANCHMEDGSA17-18	*Engraulis encrasicolus*	Mediterranean Sea	no change	stable constant	NA	NA
ANCHMEDGSA6	*Engraulis encrasicolus*	Mediterranean Sea	quadratic	stable concave	NA	NA
ANCHOBAYB	*Engraulis encrasicolus*	Celtic-Biscay Shelf	no change	stable constant	NA	NA
ANCHOSA	*Engraulis encrasicolus*	Agulhas Current	no change	stable constant	NA	NA
APOLLNSJ	*Theragra chalcogramma*	Sea of Japan	linear	decrease constant	NA	NA
APOLLPJPN	*Theragra chalcogramma*	Kuroshio Current	no change	stable constant	NA	NA
ARFLOUNDBSAI	*Atheresthes stomias*	East Bering Sea	quadratic	stable concave	NA	NA
ARFLOUNDGA	*Atheresthes stomias*	Gulf of Alaska	linear	decrease constant	NA	NA
ARFLOUNDPCOAST	*Atheresthes stomias*	California Current	linear	increase constant	NA	NA
ARGANCHONARG	*Engraulis anchoita*	Patagonian Shelf	abrupt	decrease abrupt	2000	-0.24
ARGANCHOSARG	*Engraulis anchoita*	Patagonian Shelf	no change	stable constant	NA	NA
ARGHAKENARG	*Merluccius hubbsi*	Patagonian Shelf	linear	decrease constant	NA	NA
ARGHAKESARG	*Merluccius hubbsi*	Patagonian Shelf	linear	decrease constant	NA	NA
ATKABSAI	*Pleurogrammus monopterygius*	East Bering Sea	no change	stable constant	NA	NA
AUROCKPCOAST	*Sebastes aurora*	California Current	quadratic	stable convex	NA	NA
AUSSALMONNZ	*Arripis trutta*	New Zealand Shelf	quadratic	stable concave	NA	NA
BGRDRNSWWA	*Macruronus novaezelandiae*	South West Australian Shelf	no change	stable constant	NA	NA
BGROCKPCOAST	*Sebastes melanostomus*	California Current	quadratic	stable concave	NA	NA
BIGEYECWPAC	*Thunnus obesus*	North Pacific Ocean	linear	increase constant	NA	NA
BIGHTREDSE	*Centroberyx gerrardi*	South West Australian Shelf	quadratic	stable convex	NA	NA
BLACKOREOWECR	*Allocyttus niger*	New Zealand Shelf	abrupt	increase abrupt	1980	0.01
BLACKROCKCAL	*Sebastes melanops*	California Current	abrupt	increase abrupt	2009	0.11
BLACKROCKORECOAST	*Sebastes melanops*	California Current	linear	increase constant	NA	NA
BLACKROCKWASH	*Sebastes melanops*	Gulf of Alaska	quadratic	stable concave	NA	NA
BLKMARLINIO	*Istiompax indica*	Indian Ocean	abrupt	increase abrupt	2004	0.07
BLSHARIO	*Prionace glauca*	Indian Ocean	quadratic	increase accelerated	NA	NA
BLUEFISHATLC	*Pomatomus saltatrix*	Southeast U.S. Continental Shelf	quadratic	stable convex	NA	NA
BLUEROCKCAL	*Sebastes mystinus*	California Current	linear	increase constant	NA	NA
BMACKECS	*Scomber australasicus*	East China Sea	no change	stable constant	NA	NA
BMARLINATL	*Makaira nigricans*	South Atlantic Ocean	abrupt	increase abrupt	1976	0.07
BMARLINIO	*Makaira mazara*	Indian Ocean	abrupt	increase abrupt	1989	0.1
BOCACCBCW	*Sebastes paucispinis*	Gulf of Alaska	abrupt	decrease abrupt	1982	-0.05
BOCACCSPCOAST	*Sebastes paucispinis*	California Current	linear	decrease constant	NA	NA
BRMSOJ	*Dentex tumifrons*	Sea of Japan	quadratic	stable convex	NA	NA
BRNROCKPCOAST	*Sebastes auriculatus*	California Current	quadratic	stable concave	NA	NA
BSBASSMATLC	*Centropristis striata*	Northeast U.S. Continental Shelf	linear	increase constant	NA	NA
BSBASSSATL	*Centropristis striata*	Southeast U.S. Continental Shelf	abrupt	decrease abrupt	1986	-0.16
BWHITNEA	*Micromesistius poutassou*	Barents Sea	quadratic	stable concave	NA	NA
CABEZORECOAST	*Scorpaenichthys marmoratus*	California Current	linear	increase constant	NA	NA
CABEZSCAL	*Scorpaenichthys marmoratus*	California Current	no change	stable constant	NA	NA
CALSCORPSCAL	*Scorpaena guttata*	California Current	no change	stable constant	NA	NA
CAPENOR	*Mallotus villosus*	Barents Sea	linear	decrease constant	NA	NA
CHAKESA	*Merluccius capensis*	Benguela Current	abrupt	decrease abrupt	1989	-0.07
CHILISPCOAST	*Sebastes goodei*	California Current	abrupt	decrease abrupt	2004	-0.04
CHROCKCPCOAST	*Sebastes nebulosus*	California Current	quadratic	increase accelerated	NA	NA
CHROCKNPCOAST	*Sebastes nebulosus*	Gulf of Alaska	linear	increase constant	NA	NA
CHROCKSPCOAST	*Sebastes nebulosus*	California Current	quadratic	stable concave	NA	NA
CHTRACCH	*Trachurus murphyi*	Humboldt Current	quadratic	stable concave	NA	NA
CMACKPJPN	*Scomber japonicus*	Kuroshio Current	quadratic	stable convex	NA	NA
CMACKTSST	*Scomber japonicus*	East China Sea	abrupt	decrease abrupt	1995	-0.16
COBGM	*Rachycentron canadum*	Gulf of Mexico	quadratic	stable concave	NA	NA
COD1F-XIV	*Gadus morhua*	Greenland Sea	no change	stable constant	NA	NA
COD1IN	*Gadus morhua*	Canadian Eastern Arctic - West Greenland	quadratic	stable convex	NA	NA
COD2J3KL	*Gadus morhua*	Labrador - Newfoundland	abrupt	decrease abrupt	1990	-0.43
COD3NO	*Gadus morhua*	Labrador - Newfoundland	no change	stable constant	NA	NA
COD3Pn4RS	*Gadus morhua*	Labrador - Newfoundland	abrupt	decrease abrupt	1985	-0.7
COD4TVn	*Gadus morhua*	Scotian Shelf	abrupt	decrease abrupt	1986	-0.27
COD4X5Yb	*Gadus morhua*	Northeast U.S. Continental Shelf	linear	decrease constant	NA	NA
COD5Zjm	*Gadus morhua*	Northeast U.S. Continental Shelf	abrupt	decrease abrupt	1989	-0.31
CODBA2224	*Gadus morhua*	Baltic Sea	quadratic	stable concave	NA	NA
CODBA2532	*Gadus morhua*	Baltic Sea	abrupt	decrease abrupt	1987	-0.43
CODFAPL	*Gadus morhua*	Faroe Plateau	linear	decrease constant	NA	NA
CODICE	*Gadus morhua*	Iceland Shelf and Sea	linear	decrease constant	NA	NA
CODIIIaW-IV-VIId	*Gadus morhua*	North Sea	linear	decrease constant	NA	NA
CODIS	*Gadus morhua*	Celtic-Biscay Shelf	linear	decrease constant	NA	NA
CODNEAR	*Gadus morhua*	Barents Sea	quadratic	stable convex	NA	NA
CODVIIek	*Gadus morhua*	Celtic-Biscay Shelf	no change	stable constant	NA	NA
CODVIa	*Gadus morhua*	Celtic-Biscay Shelf	quadratic	decrease decelerated	NA	NA
COWCODSCAL	*Sebastes levis*	California Current	quadratic	stable concave	NA	NA
CPRROCKPCOAST	*Sebastes caurinus*	California Current	quadratic	stable concave	NA	NA

Stock name	Species	Large Marine Ecosystem	Class	Trajectory	Shift year	Shift magnitude
CROCKPCOAST	*Sebastes pinniger*	California Current	quadratic	stable concave	NA	NA
CROCKWCVANISOGQCI	*Sebastes pinniger*	Gulf of Alaska	quadratic	stable convex	NA	NA
CSARDCSCH	*Strangomera bentincki*	Humboldt Current	quadratic	stable concave	NA	NA
CTRACSA	*Trachurus capensis*	Agulhas Current	abrupt	increase abrupt	1955	0.1
CUSKVa-XIV	*Brosme brosme*	Greenland Sea	no change	stable constant	NA	NA
DEEPCHAKESA	*Merluccius paradoxus*	Benguela Current	abrupt	increase abrupt	1962	0.23
DEEPFLATHEADSE	*Platycephalus conatus*	South West Australian Shelf	linear	increase constant	NA	NA
DKROCKPCOAST	*Sebastes crameri*	California Current	abrupt	increase abrupt	1999	0.03
DSOLEGA	*Microstomus pacificus*	Gulf of Alaska	no change	stable constant	NA	NA
DSOLEPCOAST	*Microstomus pacificus*	California Current	linear	increase constant	NA	NA
DUSROCKGA	*Sebastes variabilis*	Gulf of Alaska	quadratic	stable concave	NA	NA
EBASSIVbc-VII	*Dicentrarchus labrax*	North Sea	quadratic	stable concave	NA	NA
ECSOLESASC	*Austroglossus pectoralis*	Agulhas Current	abrupt	decrease abrupt	1999	-0.02
ESOLEHS	*Parophrys vetulus*	Gulf of Alaska	no change	stable constant	NA	NA
ESOLEPCOAST	*Parophrys vetulus*	California Current	no change	stable constant	NA	NA
FLSOLEBSAI	*Hippoglossoides elassodon*	East Bering Sea	abrupt	decrease abrupt	1991	-0.08
FLSOLEGA	*Hippoglossoides elassodon*	Gulf of Alaska	quadratic	stable convex	NA	NA
FMEG8c9a	*Lepidorhombus boscii*	Iberian Coastal	quadratic	stable convex	NA	NA
GAGSATLC	*Mycteroperca microlepis*	Southeast U.S. Continental Shelf	abrupt	increase abrupt	1978	0.16
GEMFISHNZ	*Rexea solandri*	New Zealand Shelf	abrupt	increase abrupt	1982	0.05
GEMFISHSE	*Rexea solandri*	Southeast Australian Shelf	linear	decrease constant	NA	NA
GHAL23KLMNO	*Reinhardtius hippoglossoides*	Labrador - Newfoundland	abrupt	decrease abrupt	1990	-0.2
GHALBSAI	*Reinhardtius hippoglossoides*	East Bering Sea	quadratic	stable convex	NA	NA
GOLDREDNEAR	*Sebastes norvegicus*	Barents Sea	quadratic	decrease decelerated	NA	NA
GOLDREDV-VI-XII-XIV	*Sebastes norvegicus*	Greenland Sea	abrupt	decrease abrupt	1979	-0.06
GOPHERSPCOAST	*Sebastes carnatus*	California Current	quadratic	stable concave	NA	NA
GRAMBERGM	*Seriola dumerili*	Gulf of Mexico	quadratic	stable concave	NA	NA
GRAMBERSATLC	*Seriola dumerili*	Southeast U.S. Continental Shelf	quadratic	stable concave	NA	NA
GRNSTROCKPCOAST	*Sebastes elongatus*	California Current	quadratic	increase accelerated	NA	NA
GRSPROCKNCAL	*Sebastes chlorostictus*	California Current	quadratic	increase decelerated	NA	NA
GRSPROCKSCAL	*Sebastes chlorostictus*	California Current	abrupt	increase abrupt	1972	0.02
GTRIGGM	*Balistes capriscus*	Gulf of Mexico	quadratic	stable concave	NA	NA
HAD4X5Y	*Melanogrammus aeglefinus*	Northeast U.S. Continental Shelf	no change	stable constant	NA	NA
HAD5Y	*Melanogrammus aeglefinus*	Northeast U.S. Continental Shelf	linear	decrease constant	NA	NA
HADFAPL	*Melanogrammus aeglefinus*	Faroe Plateau	linear	decrease constant	NA	NA
HADICE	*Melanogrammus aeglefinus*	Iceland Shelf and Sea	no change	stable constant	NA	NA
HADIS	*Melanogrammus aeglefinus*	Celtic-Biscay Shelf	no change	stable constant	NA	NA
HADNEAR	*Melanogrammus aeglefinus*	Barents Sea	abrupt	increase abrupt	2005	0.2
HADNS-IIIa-VIa	*Melanogrammus aeglefinus*	North Sea	abrupt	decrease abrupt	1999	-0.24
HADROCK	*Melanogrammus aeglefinus*	Celtic-Biscay Shelf	quadratic	stable convex	NA	NA
HADVIIb-k	*Melanogrammus aeglefinus*	Celtic-Biscay Shelf	no change	stable constant	NA	NA
HAKENRTN	*Merluccius merluccius*	Celtic-Biscay Shelf	abrupt	increase abrupt	2007	0.6
HAKESOTH	*Merluccius merluccius*	Iberian Coastal	abrupt	increase abrupt	2004	0.35
HAKESPPSAF	*Merluccius spp*	Benguela Current	linear	increase constant	NA	NA
HERR2224IIIa	*Clupea harengus*	North Sea	abrupt	decrease abrupt	2001	-0.27
HERR2529-32	*Clupea harengus*	Baltic Sea	abrupt	decrease abrupt	1981	-0.1
HERR30-31	*Clupea harengus*	Baltic Sea	quadratic	stable convex	NA	NA
HERR4RSP	*Clupea harengus*	Labrador - Newfoundland	no change	stable constant	NA	NA
HERR4VWX	*Clupea harengus*	Scotian Shelf	linear	decrease constant	NA	NA
HERRCC	*Clupea pallasii*	Gulf of Alaska	linear	decrease constant	NA	NA
HERRHG	*Clupea pallasii*	Gulf of Alaska	abrupt	decrease abrupt	1982	-0.27
HERRIsum	*Clupea harengus*	Iceland Shelf and Sea	abrupt	decrease abrupt	2008	-0.27
HERRNIRS	*Clupea harengus*	Celtic-Biscay Shelf	no change	stable constant	NA	NA
HERRNORSS	*Clupea harengus*	Norwegian Sea	quadratic	stable concave	NA	NA
HERRNS-IIIa-VIId	*Clupea harengus*	North Sea	no change	stable constant	NA	NA
HERRNWATLC	*Clupea harengus*	Northeast U.S. Continental Shelf	no change	stable constant	NA	NA
HERRPRD	*Clupea pallasii*	Gulf of Alaska	abrupt	decrease abrupt	1964	-0.36
HERRRIGA	*Clupea harengus*	Baltic Sea	linear	increase constant	NA	NA
HERRSIRS	*Clupea harengus*	Celtic-Biscay Shelf	no change	stable constant	NA	NA
HERRSOG	*Clupea pallasii*	Gulf of Alaska	abrupt	decrease abrupt	1963	-0.21
HERRVIaVIIbc	*Clupea harengus*	Celtic-Biscay Shelf	linear	decrease constant	NA	NA
HERRWCVANI	*Clupea pallasii*	Gulf of Alaska	linear	decrease constant	NA	NA
HMACKIIa-IVa-Vb-VIa-VII-VIII	*Trachurus trachurus*	Norwegian Sea	quadratic	stable convex	NA	NA
JANCHOPJPN	*Engraulis japonicus*	Kuroshio Current	quadratic	stable concave	NA	NA
JANCHOSETO	*Engraulis japonicus*	Kuroshio Current	no change	stable constant	NA	NA
JANCHOTSST	*Engraulis japonicus*	East China Sea	quadratic	stable concave	NA	NA
JMACKPJPN	*Trachurus japonicus*	Kuroshio Current	quadratic	stable concave	NA	NA
JMACKTSST	*Trachurus japonicus*	East China Sea	abrupt	increase abrupt	1985	0.24
KELPGREENLINGORECOAST	*Hexagrammos decagrammus*	California Current	no change	stable constant	NA	NA
LINGCODNPCOAST	*Ophiodon elongatus*	California Current	no change	stable constant	NA	NA
LINGCODSPCOAST	*Ophiodon elongatus*	California Current	linear	increase constant	NA	NA
LINGVa	*Molva molva*	Iceland Shelf and Sea	quadratic	increase accelerated	NA	NA
LNOSESKAPCOAST	*Raja rhina*	California Current	linear	increase constant	NA	NA
LSTHORNHPCOAST	*Sebastolobus altivelis*	California Current	quadratic	increase decelerated	NA	NA
MACKNEICES	*Scomber scombrus*	Norwegian Sea	linear	increase constant	NA	NA
MACKNWATLSA3-4	*Scomber scombrus*	Labrador - Newfoundland	no change	stable constant	NA	NA
MEG8c9a	*Lepidorhombus whiffiagonis*	Iberian Coastal	quadratic	stable convex	NA	NA
MEGVII-VIIIabd	*Lepidorhombus whiffiagonis*	Celtic-Biscay Shelf	quadratic	stable convex	NA	NA
MENATGM	*Brevoortia patronus*	Gulf of Mexico	no change	stable constant	NA	NA
MENATLAN	*Brevoortia tyrannus*	Northeast U.S. Continental Shelf	no change	stable constant	NA	NA
MONKGOMNGB	*Lophius americanus*	Northeast U.S. Continental Shelf	quadratic	stable concave	NA	NA
MONKSGBMATL	*Lophius americanus*	Northeast U.S. Continental Shelf	abrupt	increase abrupt	1992	0.05
MORWONGESE	*Nemadactylus macropterus*	Southeast Australian Shelf	abrupt	decrease abrupt	1984	-0.09
MORWONGWSE	*Nemadactylus macropterus*	South West Australian Shelf	quadratic	increase accelerated	NA	NA

Stock name	Species	Large Marine Ecosystem	Class	Trajectory	Shift year	Shift magnitude
MUTSNAPSATLCGM	*Lutjanus analis*	Gulf of Mexico	linear	decrease constant	NA	NA
NPOUTIIIa-IV	*Trisopterus esmarkii*	North Sea	no change	stable constant	NA	NA
NROCKBSAI	*Sebastes polyspinis*	East Bering Sea	quadratic	stable concave	NA	NA
NROCKGA	*Sebastes polyspinis*	Gulf of Alaska	quadratic	stable concave	NA	NA
NRSOLEEBSAI	*Lepidopsetta polyxystra*	East Bering Sea	quadratic	stable concave	NA	NA
NRSOLEGA	*Lepidopsetta polyxystra*	Gulf of Alaska	quadratic	stable concave	NA	NA
NZLINGESE	*Genypterus blacodes*	Southeast Australian Shelf	abrupt	increase abrupt	1990	0.04
NZLINGLIN6b	*Genypterus blacodes*	South Pacific Ocean	no change	stable constant	NA	NA
NZLINGLIN72	*Genypterus blacodes*	New Zealand Shelf	linear	increase constant	NA	NA
NZLINGLIN7WC	*Genypterus blacodes*	New Zealand Shelf	quadratic	stable convex	NA	NA
NZLINGWSE	*Genypterus blacodes*	South West Australian Shelf	no change	stable constant	NA	NA
OFLOUNECS	*Paralichthys olivaceus*	East China Sea	abrupt	decrease abrupt	1995	-0.13
OROUGHYSE	*Hoplostethus atlanticus*	South West Australian Shelf	abrupt	increase abrupt	1988	0.04
PACBTUNA	*Thunnus orientalis*	South Pacific Ocean	abrupt	decrease abrupt	1958	-0.24
PANCHCCH	*Engraulis ringens*	Humboldt Current	no change	stable constant	NA	NA
PANCHNCHSP	*Engraulis ringens*	Humboldt Current	no change	stable constant	NA	NA
PANCHPERUNC	*Engraulis ringens*	Humboldt Current	no change	stable constant	NA	NA
PATGRENADIERCH	*Macruronus magellanicus*	Humboldt Current	linear	decrease constant	NA	NA
PATGRENADIERSARG	*Macruronus magellanicus*	Patagonian Shelf	abrupt	increase abrupt	1993	0.11
PCEELARGS	*Genypterus blacodes*	Patagonian Shelf	abrupt	increase abrupt	1996	0.06
PCEELCSCH	*Genypterus blacodes*	Humboldt Current	linear	decrease constant	NA	NA
PCEELSCH	*Genypterus blacodes*	Humboldt Current	linear	decrease constant	NA	NA
PCOD5ABCD	*Gadus macrocephalus*	Gulf of Alaska	no change	stable constant	NA	NA
PCODAI	*Gadus macrocephalus*	Aleutian Islands	quadratic	stable concave	NA	NA
PCODBS	*Gadus macrocephalus*	East Bering Sea	quadratic	stable convex	NA	NA
PCODGA	*Gadus macrocephalus*	Gulf of Alaska	linear	increase constant	NA	NA
PCODWCVANI	*Gadus macrocephalus*	Gulf of Alaska	no change	stable constant	NA	NA
PERCHEBSAI	*Sebastes alutus*	East Bering Sea	abrupt	increase abrupt	1982	0.04
PERCHHG	*Sebastes alutus*	Gulf of Alaska	abrupt	decrease abrupt	1963	-0.03
PERCHWCVANI	*Sebastes alutus*	Gulf of Alaska	abrupt	increase abrupt	1964	0.03
PHAKEPCOAST	*Merluccius productus*	California Current	no change	stable constant	NA	NA
PILCHPJPN	*Sardinops melanostictus*	Kuroshio Current	abrupt	decrease abrupt	1986	-0.63
PILCHTSST	*Sardinops melanostictus*	East China Sea	abrupt	decrease abrupt	1987	-0.41
PLAIC7d	*Pleuronectes platessa*	Celtic-Biscay Shelf	no change	stable constant	NA	NA
PLAICCELT	*Pleuronectes platessa*	Celtic-Biscay Shelf	linear	decrease constant	NA	NA
PLAICECHW	*Pleuronectes platessa*	Celtic-Biscay Shelf	no change	stable constant	NA	NA
PLAICIS	*Pleuronectes platessa*	Celtic-Biscay Shelf	linear	decrease constant	NA	NA
PLAICNS	*Pleuronectes platessa*	North Sea	quadratic	stable concave	NA	NA
POLL5YZ	*Pollachius virens*	Northeast U.S. Continental Shelf	no change	stable constant	NA	NA
POLLFAPL	*Pollachius virens*	Faroe Plateau	no change	stable constant	NA	NA
POLLIEG	*Pollachius virens*	Iceland Shelf and Sea	no change	stable constant	NA	NA
POLLNEAR	*Pollachius virens*	Barents Sea	no change	stable constant	NA	NA
POLLNS-VI-IIIa	*Pollachius virens*	North Sea	quadratic	decrease decelerated	NA	NA
POPERCHGA	*Sebastes alutus*	Gulf of Alaska	quadratic	stable convex	NA	NA
POPERCHPCOAST	*Sebastes alutus*	California Current	abrupt	increase abrupt	2009	0.05
PSOLEPCOAST	*Eopsetta jordani*	California Current	no change	stable constant	NA	NA
PTOOTHFISHMI	*Dissostichus eleginoides*	South Pacific Ocean	abrupt	increase abrupt	1998	0.03
RBRMECS	*Pagrus major*	East China Sea	abrupt	increase abrupt	1991	0.03
RBRMSETOE	*Pagrus major*	Kuroshio Current	linear	increase constant	NA	NA
RBRMSETOW	*Pagrus major*	Kuroshio Current	quadratic	stable concave	NA	NA
REDFEAUS	*Centroberyx affinis*	Southeast Australian Shelf	linear	decrease constant	NA	NA
REDFISHSPP3M	*Sebastes spp*	Labrador - Newfoundland	abrupt	decrease abrupt	2005	-0.46
REXSOLEGA	*Glyptocephalus zachirus*	Gulf of Alaska	quadratic	stable concave	NA	NA
REYEROCKBSAI	*Sebastes aleutianus*	East Bering Sea	abrupt	increase abrupt	2003	0.09
REYEROCKGA	*Sebastes aleutianus*	Gulf of Alaska	abrupt	increase abrupt	1991	0.01
RGROUPSATL	*Epinephelus morio*	Southeast U.S. Continental Shelf	quadratic	stable convex	NA	NA
RHERRTSST	*Etrumeus teres*	Sea of Japan	no change	stable constant	NA	NA
RPORGYSATLC	*Pagrus pagrus*	Southeast U.S. Continental Shelf	no change	stable constant	NA	NA
RSNAPGM	*Lutjanus campechanus*	Gulf of Mexico	quadratic	increase accelerated	NA	NA
RSNAPSATLC	*Lutjanus campechanus*	Southeast U.S. Continental Shelf	linear	increase constant	NA	NA
RSOLEGA	*Lepidopsetta bilineata*	Gulf of Alaska	linear	decrease constant	NA	NA
RSOLEHSTR	*Lepidopsetta bilineata*	Gulf of Alaska	no change	stable constant	NA	NA
RSROCKBCWN	*Sebastes proriger*	Gulf of Alaska	linear	increase constant	NA	NA
RSROCKBCWS	*Sebastes proriger*	Gulf of Alaska	linear	increase constant	NA	NA
SABLEFEBSAIGA	*Anoplopoma fimbria*	Gulf of Alaska	no change	stable constant	NA	NA
SABLEFPCAN	*Anoplopoma fimbria*	Gulf of Alaska	abrupt	increase abrupt	1977	0.05
SABLEFPCOAST	*Anoplopoma fimbria*	California Current	quadratic	stable concave	NA	NA
SAILEATL	*Istiophorus albicans*	South Atlantic Ocean	quadratic	stable concave	NA	NA
SAILWATL	*Istiophorus albicans*	South Atlantic Ocean	linear	increase constant	NA	NA
SARDMEDGSA17-18	*Sardina pilchardus*	Mediterranean Sea	quadratic	stable convex	NA	NA
SARDPVIIIc-IXa	*Sardina pilchardus*	Iberian Coastal	linear	decrease constant	NA	NA
SARDSAS	*Sardinops sagax*	Agulhas Current	abrupt	decrease abrupt	2001	-0.53
SARDSAW	*Sardinops sagax*	Benguela Current	no change	stable constant	NA	NA
SARDWSE	*Sardinops sagax*	South West Australian Shelf	abrupt	increase abrupt	1998	0.22
SAURNWPAC	*Cololabis saira*	Kuroshio Current	no change	stable constant	NA	NA
SBARSHARATL	*Carcharhinus plumbeus*	Gulf of Mexico	quadratic	stable convex	NA	NA
SBELLYROCKPCOAST	*Sebastes jordani*	California Current	quadratic	stable concave	NA	NA
SBT	*Thunnus maccoyii*	Indian Ocean	linear	increase constant	NA	NA
SBWHITARGS	*Micromesistius australis*	Patagonian Shelf	quadratic	stable convex	NA	NA
SBWHITCH	*Micromesistius australis*	Humboldt Current	quadratic	stable convex	NA	NA
SEELNSSA1	*Ammodytes spp*	North Sea	no change	stable constant	NA	NA
SEELNSSA2	*Ammodytes spp*	North Sea	abrupt	decrease abrupt	1996	-0.52
SEELNSSA3	*Ammodytes spp*	North Sea	no change	stable constant	NA	NA
SHCROCKPCOAST	*Sebastes zacentrus*	California Current	no change	stable constant	NA	NA

Stock name	Species	Large Marine Ecosystem	Class	Trajectory	Shift year	Shift magnitude
SILVERFISHSE	*Seriolella punctata*	Southeast Australian Shelf	quadratic	stable concave	NA	NA
SKJCIO	*Katsuwonus pelamis*	Indian Ocean	abrupt	increase abrupt	1989	0.32
SKJCWPAC	*Katsuwonus pelamis*	North Pacific Ocean	linear	increase constant	NA	NA
SKJEATL	*Katsuwonus pelamis*	South Atlantic Ocean	linear	increase constant	NA	NA
SKJWATL	*Katsuwonus pelamis*	North Atlantic Ocean	abrupt	increase abrupt	1980	0.55
SMDOGATL	*Mustelus canis*	Southeast U.S. Continental Shelf	quadratic	stable concave	NA	NA
SNAPSAUSNSG	*Chrysophrys auratus*	South West Australian Shelf	linear	increase constant	NA	NA
SNAPSAUSSGSV	*Chrysophrys auratus*	South West Australian Shelf	linear	increase constant	NA	NA
SNAPSAUSSSG	*Chrysophrys auratus*	South West Australian Shelf	quadratic	stable concave	NA	NA
SNOWGROUPSATLC	*Epinephelus niveatus*	Southeast U.S. Continental Shelf	no change	stable constant	NA	NA
SNROCKPCOAST	*Sebastes diploproa*	California Current	abrupt	increase abrupt	2001	0.08
SOLECS	*Solea solea*	Celtic-Biscay Shelf	no change	stable constant	NA	NA
SOLEIIIa-2224	*Solea solea*	North Sea	linear	decrease constant	NA	NA
SOLEIS	*Solea solea*	Celtic-Biscay Shelf	linear	decrease constant	NA	NA
SOLENS	*Solea solea*	North Sea	no change	stable constant	NA	NA
SOLEVIIIab	*Solea solea*	Celtic-Biscay Shelf	linear	decrease constant	NA	NA
SOLEVIId	*Solea solea*	Celtic-Biscay Shelf	no change	stable constant	NA	NA
SOLEVIIe	*Solea solea*	Celtic-Biscay Shelf	abrupt	increase abrupt	1975	0.1
SOUTHHAKECH	*Merluccius australis*	Humboldt Current	quadratic	stable concave	NA	NA
SPANMACKGM	*Scomberomorus maculatus*	Gulf of Mexico	no change	stable constant	NA	NA
SPANMACKSATLC	*Scomberomorus maculatus*	Southeast U.S. Continental Shelf	linear	increase constant	NA	NA
SPANMACKSETO	*Scomberomorus niphonius*	Kuroshio Current	quadratic	stable convex	NA	NA
SPHAKECH	*Merluccius gayi*	Humboldt Current	abrupt	decrease abrupt	1995	-0.07
SPRAT22-32	*Sprattus sprattus*	Baltic Sea	abrupt	increase abrupt	1987	0.19
SPSDOGPCOAST	*Squalus suckleyi*	California Current	linear	increase constant	NA	NA
SSTHORNHGA	*Sebastolobus alascanus*	Gulf of Alaska	no change	stable constant	NA	NA
SSTHORNHPCOAST	*Sebastolobus alascanus*	California Current	abrupt	decrease abrupt	1971	0
STFLOUNNPCOAST	*Platichthys stellatus*	California Current	abrupt	decrease abrupt	1987	-0.29
STFLOUNSPCOAST	*Platichthys stellatus*	California Current	no change	stable constant	NA	NA
STMARLINIO	*Kajikia audax*	Indian Ocean	quadratic	stable concave	NA	NA
STMARLINNEPAC	*Kajikia audax*	North Pacific Ocean	no change	stable constant	NA	NA
STMARLINSWPO	*Kajikia audax*	South Pacific Ocean	quadratic	stable concave	NA	NA
STMARLINWCNPAC	*Kajikia audax*	North Pacific Ocean	linear	decrease constant	NA	NA
SWHITSE	*Sillago flindersi*	Southeast Australian Shelf	abrupt	increase abrupt	1983	0.1
SWORDEPAC	*Xiphias gladius*	South Pacific Ocean	linear	increase constant	NA	NA
SWORDNATL	*Xiphias gladius*	North Atlantic Ocean	linear	increase constant	NA	NA
SWORDNPAC	*Xiphias gladius*	North Pacific Ocean	quadratic	stable concave	NA	NA
TIGERFLATSE	*Neoplatycephalus richardsoni*	Southeast Australian Shelf	linear	increase constant	NA	NA
TILEGM	*Lopholatilus chamaeleonticeps*	Gulf of Mexico	linear	increase constant	NA	NA
TILESATLC	*Lopholatilus chamaeleonticeps*	Southeast U.S. Continental Shelf	abrupt	increase abrupt	1985	0.08
TREVALLYTRE7	*Pseudocaranx dentex*	New Zealand Shelf	linear	increase constant	NA	NA
VSNAPGM	*Rhomboplites aurorubens*	Gulf of Mexico	quadratic	increase accelerated	NA	NA
VSNAPSATLC	*Rhomboplites aurorubens*	Southeast U.S. Continental Shelf	abrupt	increase abrupt	1980	0.1
WANGLVII-VIIIabd	*Lophius piscatorius*	Celtic-Biscay Shelf	linear	increase constant	NA	NA
WANGLVIIIc-IXa	*Lophius piscatorius*	Iberian Coastal	quadratic	decrease decelerated	NA	NA
WHAKE4T	*Urophycis tenuis*	Scotian Shelf	abrupt	decrease abrupt	1983	-0.26
WHAKEGBGOM	*Urophycis tenuis*	Northeast U.S. Continental Shelf	no change	stable constant	NA	NA
WHITNS-VIId	*Merlangius merlangus*	North Sea	linear	decrease constant	NA	NA
WHITVIIa	*Merlangius merlangus*	Celtic-Biscay Shelf	quadratic	decrease decelerated	NA	NA
WHITVIa	*Merlangius merlangus*	Celtic-Biscay Shelf	no change	stable constant	NA	NA
WMARLINATL	*Tetrapturus albidus*	South Atlantic Ocean	quadratic	stable concave	NA	NA
WPOLLAI	*Theragra chalcogramma*	Aleutian Islands	quadratic	stable convex	NA	NA
WPOLLBOGO	*Theragra chalcogramma*	East Bering Sea	no change	stable constant	NA	NA
WPOLLEBS	*Theragra chalcogramma*	East Bering Sea	no change	stable constant	NA	NA
WPOLLGA	*Theragra chalcogramma*	Gulf of Alaska	abrupt	decrease abrupt	1981	-0.16
WPOLLNSO	*Theragra chalcogramma*	Sea of Okhotsk	abrupt	decrease abrupt	1995	-0.07
WPOLLWBS	*Theragra chalcogramma*	West Bering Sea	abrupt	decrease abrupt	1987	-0.14
WROCKPCOAST	*Sebastes entomelas*	California Current	quadratic	stable concave	NA	NA
YEGROUPGM	*Epinephelus flavolimbatus*	Gulf of Mexico	no change	stable constant	NA	NA
YELLGB	*Limanda ferruginea*	Northeast U.S. Continental Shelf	quadratic	stable convex	NA	NA
YELLSNEMATL	*Limanda ferruginea*	Northeast U.S. Continental Shelf	abrupt	decrease abrupt	1987	-0.75
YEYEROCKGA	*Sebastes ruberrimus*	Gulf of Alaska	no change	stable constant	NA	NA
YEYEROCKPCOAST	*Sebastes ruberrimus*	California Current	abrupt	increase abrupt	1976	0.02
YFINCWPAC	*Thunnus albacares*	North Pacific Ocean	linear	increase constant	NA	NA
YNOSESKACSCH	*Zearaja chilensis*	Humboldt Current	abrupt	decrease abrupt	2001	-0.01
YSOLEBSAI	*Limanda aspera*	East Bering Sea	quadratic	stable concave	NA	NA
YTROCKNPCOAST	*Sebastes flavidus*	California Current	quadratic	stable concave	NA	NA
YTSNAPSATLCGM	*Ocyurus chrysurus*	Gulf of Mexico	no change	stable constant	NA	NA

Table S2: Hierarchical partitioning and randomization test outputs for negative PAS.

variable	I[a]	J[b]	Total	Z.score[c]
Age_maturity	0.25	-0.23	0.03	-0.37
Length_maturity	0.58	0.11	0.69	0.09
DemersPelag	0.16	-0.15	0.01	-0.5
SST_mean	1.83	0.32	2.15	**1.85**
SST_change	5.68	0.51	6.19	**6.59**
ER_mean	0.04	-0.02	0.02	-0.65
ER_change	0.23	0.27	0.5	-0.39
coord_X	0.12	-0.08	0.04	-0.56
coord_Y	0.15	0.07	0.22	-0.52

[a] Independent contribution of the predictor to explained variance.
[b] Joint contribution to explained variance.
[c] Independent contribution Z-score from 999 randomizations. Values in bold indicate statistical significance based on upper 0.95 confidence limit.

Table S3: Hierarchical partitioning and randomization test outputs for positive PAS.

variable	I[a]	J[b]	Total	Z.score[c]
Age_maturity	3.42	0.77	4.18	**4.17**
Length_maturity	0.51	1.05	1.56	-0.01
DemersPelag	0.32	0.03	0.35	-0.25
SST_mean	0.33	0.51	0.84	-0.29
SST_change	2.5	0.79	3.29	**2.65**
ER_mean	2.43	1.18	3.61	**2.66**
ER_change	5.93	0.72	6.65	**6.91**
coord_X	1.64	-0.37	1.26	1.55
coord_Y	1.58	2.41	3.99	1.36

[a] Independent contribution of the predictor to explained variance.

[b] Joint contribution to explained variance.

[c] Independent contribution Z-score from 999 randomizations. Values in bold indicate statistical significance based on upper 0.95 confidence limit.
